# Activity constraints and the emergence of non-scale-free networks: Evidence from hip-hop and academia

**DOI:** 10.1371/journal.pone.0345862

**Published:** 2026-03-30

**Authors:** Jaemin Lee, Yujie Li

**Affiliations:** 1 Department of Sociology, The Chinese University of Hong Kong, Sha Tin, New Territories, Hong Kong SAR, Hong Kong, China; 2 Division of Computational Social Science, The Chinese University of Hong Kong, Shenzhen, Shenzhen, Guangdong, China; Beijing University of Technology, CHINA

## Abstract

Power-law distributions are widely used to characterize complex networks, yet recent work shows that scale-freeness is far less universal than once assumed, especially in social networks. We propose activity constraints—the finite time and effort required to complete collaborative ties—as a mechanism that limits hub growth and produces non-scale-free degree distributions. Using synthetic models and mathematical derivations, we show that imposing capacity limits on high-quality actors shifts degree distributions away from power laws and toward log-normal forms. We then examine degree saturation in two empirical settings: hip-hop featuring networks and academic coauthorship networks. In both cases, high-quality actors receive more ties, but their realized degree saturates relative to expectations, yielding sublinear or S-shaped quality-degree relationships. These findings demonstrate how micro-level workload constraints generate macro-level deviations from scale-free structure and highlight the importance of distinguishing activity-intensive from low-cost tie-formation processes when evaluating claims of scale-freeness in networks.

## Introduction

The property of power laws to understand and characterize the degree distribution of a network is widespread across various disciplines. Generally, a network is deemed scale-free if the fraction of nodes with degree *k* follows a power law $k^{-\alpha }$. Ever since the seminal paper [1] finding the distribution of hyper-links in the World Wide Web (WWW) is scale-free, there has been an explosion of research reporting such cases in many empirical networks [2]. The adjudication of whether degree distributions follow a power law and exhibit scale-freeness has significant implications across various fields. Recognizing degree properties and influential nodes helps guide strategies for cybersecurity and infrastructure resilience [3,4] and optimize resource allocation for better information dissemination and immunization strategies [5].

A more recent body of work suggests that one should be cautious about assuming away the universality of scale-free distributions. Stumpf and Porter [6] argue that many of the systems that are characterized as having a power law distribution could equally come from another heavy-tailed distribution. Broido and Clauset [7] investigated a wide array of network data to claim that scale-free patterns are empirically varied, although their taxonomy that goes from “weakest” to “strongest” scale-freeness has been contested since [8–10]. Notably, it was shown that a degree of power-law distribution is stronger in technological and biological networks than in social networks. That is, for social networks, scale-free is often not a better model than a non-scale-free but heavy-tail distribution such as a log-normal distribution. Parallel evidence comes from research on sexual networks. Hamilton, Handcock, and Morris [11] show that power-law models not only fit poorly to US sexual partnership data but also yield misleading epidemic predictions, while Handcock and Jones [12] demonstrate that negative binomial and other finite-variance models often outperform power laws across multiple populations. These studies underline that the assumption of infinite variance is rarely warranted in human social networks, echoing the broader point that scale-free structure is far less universal than once claimed.

In case social networks do not exhibit scale-free distributions than usually assumed, what explains such patterns? Broido and Clauset note that “less is known about mechanisms that produce non-scale-free structural patterns.” (p.8) [7] We address this gap by proposing activity constraints as a mechanism that limits hub growth in social networks. Classical sociological work [13,14] emphasizes that forming and sustaining social ties requires non-trivial investments of time, and emotional energy, and Dunbar’s number [15] highlights the cognitive limits on maintaining stable social relationships. These insights imply that, unlike technological networks where links are effectively costless, social actors face finite collaborative capacity.

Our argument also echoes ideas in transaction cost theory: collaboration entails coordination and effort costs that accumulate as actors take on more partners. Whereas market transaction costs emphasize search, bargaining, and enforcement [16,17], activity constraints capture the workload saturation inherent in social ties, particularly in collaboration-intensive settings. High-quality actors may attract many potential ties, but their limited time and energy impose a natural ceiling on realized degree.

Many of the social networks examined by Broido and Clauset likely operate under such constraints. Academic authors cannot contribute meaningfully to unlimited coauthorships; film and novel writers avoid over centralizing characters; and leaders in clandestine organizations avoid excessive connectivity to reduce exposure risk. These examples suggest that growth constraints are not incidental but structural, shaping how degree distributions deviate from scale-free forms. Building on this intuition, we extend the fitness-corrected Barabási-Albert model [18,19] to show how self-regulated limits on tie completion can generate non-scale-free, often log-normal, degree distributions.

Our analysis is twofold: formal and empirical modelling. First, we conduct a numerical experiment using synthetic networks created by a linear preferential attachment model [1,20] and see how imposing an upper bound of accepting ties impacts the overall degree distribution. We then provide mathematical derivations regarding why the degree distributions shaped by the proposed activity constraint processes necessarily deviate from scale-free properties.

Second, do social hubs exhibit degree saturation? We approach this question by empirically investigating the functional form of the dependency between quality and degree in the domains of hip-hop music production and academic publication. In both domains, collaboration is a general form among actors: They often “feature” on another artist’s song or “co-author” an academic paper with other researchers. At the same time, actors in these domains likely face a physical upper bound in completing ties: Hip-hop artists should commit to producing a song together and researchers are obligated to contribute significantly to claim authorship.

We first leverage the empirical context of a collaboration network of artists in the Korean hip-hop industry—a setting where “featuring” among independent artists is a general form of song production and tie formation is driven by the hierarchy of artists’ quality and status [21–25]. Are featuring invites disproportionately concentrated on a few star rappers, and do they exhibit k(in) higher or lower than expected by quality? Our large-scale data collection allows us to estimate the functional form of the effect of the artist’s quality on the featuring behavior. For the network of academic collaboration, we extract and analyze a large-scale data lake, SciSciNet, that encompasses 134M scientific publications over 19 different fields [26]. Our focus here is whether coauthor degree distribution, as opposed to that of citation degree, tends to be non-scale-free and, if so, whether co-author degree increases with author quality in a sublinear manner, indicating degree saturation of high-quality researchers.

We find that imposing an upper bound of accepting ties makes the preferential attachment mechanism more likely to produce a log-normal degree distribution rather than a scale-free degree distribution. Empirically, in a hip-hop collaboration network that shows a non-scale-free degree distribution, quality has a sub-linear relationship with *k*^(*in*)^: Quality increases with more received ties but not at the level expected by their quality, especially top-level rappers. The academic collaboration networks across various disciplines also fit a log-normal degree distribution better than a power law while the citation degree distribution generally approximates a power law. Similarly to the hip-hop context, we find an S-shaped non-linear effect of author quality (H-Index) on coauthor degree. These findings add yet another evidence for the heterogeneity of scale-free networks [7] and contribute a mechanism that could be unique for activity-based social networks. Conversely, this mechanism suggests that it is essential to also consider network data that do not impose activity constraints (e.g., following relationship social media) when ascertaining a power law for social networks. This article significantly extends our previous conference proceeding [27] by expanding the theory of activity constraints in tie formation, offering mathematical derivations that support the numerical simulations, and adding academic collaboration networks as a new empirical test bed.

### Related work on mechanisms of non-scale-free structure

The phenomenon we address is the tendency of social networks to deviate from scale-free degree distributions. We theorize this deviation as an activity constraint—in practice, a form of collaborative workload saturation that limits the number of ties an actor can sustain. Prior studies have proposed several mechanisms that suppress hub growth in preferential attachment processes.

Beyond the debate over scale-free versus non-scale-free distributions, recent studies have documented more complex structural regularities in networks, including multifractal generating measures [28], hidden generative rules [29], hierarchical growth in neural systems [29], geometric renormalization [30], reconstruction under adversarial interventions [31], and controllability constraints [32]. These works collectively demonstrate that real networks often deviate from simple scale-free forms, underscoring the need to identify mechanisms that account for such deviations. In network science, several models have been proposed to suppress hub growth within preferential attachment processes. Site aging models [33,34] assume that a node’s attractiveness decays with time, such that older nodes gradually stop receiving new links. Finite memory models [35] instead posit that only a limited set of recent nodes are eligible for attachment, which increases clustering and reduces the dominance of old hubs. Vertex deactivation models [36] and related active–inactive frameworks assume that nodes eventually stop participating altogether, producing truncated heavy-tailed distributions.

Our proposed activity-constraint model shares the spirit of these approaches in that it limits the growth of hubs, but it differs in the triggering mechanism: rather than time, memory, or stochastic state changes, we focus on degree-linked workload saturation. This mechanism is particularly well-suited to activity-intensive social ties, such as coauthoring or musical collaboration, where the cost of each tie is substantial. Our contribution is therefore complementary. While prior work highlights structural, temporal, or probabilistic constraints on growth, we highlight activity constraints as a behavioral mechanism that explains why collaboration networks in particular deviate from scale-free forms.

## Preferential attachment with activity constrains

### Preliminaries

We follow [2,7] for the definitions of scale-free networks and empirical testing against alternative heavy-tailed distributions. For the degree sequence $\{k_{i}\}=k_{1},k_{2},\cdots ,k_{n}$ of a given network data set, we estimate the best-fitting power-law distribution of the form


$$Pr(k)=Ck^{-\alpha },k\geq k_{min}\geq 1$$ (1)

Where α is the scaling exponent, *C* is the normalizing constant, and *k* is integer valued. This specification models only the distribution’s upper tail, i.e., degree values $k\geq k_{min}$, and discards data from any non-power-law portion in the lower distribution.

The Barabási-Albert model specifies growth and preferential attachment as a probabilistic mechanism. The probability Π(k) that a link of the new node connects to node *i* depends on the degree *k*_*i*_ as


$$\Pi (k_{i})=\frac{k_{i}}{\sum_{j}k_{j}}$$ (2)

Eq 2 implies that if a new node chooses between a *k* = 1 node and a *k* = 3 node, it is three times as likely to connect to the latter node. Due to preferential attachment, the larger nodes acquire links at the expense of the smaller nodes. $\Pi (k_{i})$ needs to depend on the degrees for the resulting network to have a pure power law for *Pr*(*k*). While sublinear preferential attachment (α<1) limits the size and number of hubs and induces a stretched exponential distribution, linear or super-linear (α>1) preferential attachment predicts the emergence of the rich-gets-richer processes for the degree distribution.

### Activity constraints in tie formation: Theory

According to Granovetter [13], tie strength is defined as “a (probably linear) combination of the amount of time, emotional intensity, the intimacy, and reciprocal services which characterize the tie.” (p. 1361) More precisely, his seminal study on job search contacts measured how often respondents interacted with their contact—often (twice a week), occasionally (more than once a week), and rarely (once a year or less). The strength of a tie—or the foundation upon which social ties are built and sustained among individuals—is therefore a product of invested time and effort, often shaped by shared interests, proximity, and repeated interactions.

The amount of time and effort required, however, may well vary depending on what comprises a tie. It was acquaintanceship in Granovetter’s job-search study, in which he found those who are not very close are found to be most helpful for someone to get a job. Simple encounters or brief greetings may be sufficient to establish acquaintanceship. Acquaintanceship in everyday life typically does not function for the instrumental purpose but, when specific needs, such as job searches, arise, can be transformed into social capital as resources. In addition to acquaintanceship, social ties can consist of virtual, non-face-to-face relationships. Examples include social media follows or citations among scholars. In this case, tie formation requires a minimal level of time and energy: Being followed or cited by others is generally not burdensome for people, as the commitment to form such ties is lighter than encounters or greetings in an acquaintance network.

By contrast, sociological research has long emphasized that humans face cognitive and temporal limits in sustaining more demanding relationships. Dunbar [15] famously argued that there is a ceiling on the number of stable social ties individuals can maintain, while ego-network surveys consistently find only a handful of close confidants [37–39]. Classic theories of tie strength [13], social foci [40], and cumulative advantage [41] all implicitly recognize that effortful ties are costly, and Simmel’s early account of urban life already noted the burden of excessive contacts [42]. More recent studies of communication networks show that individuals have a finite “social capacity” and reallocate attention when new ties are added [43,44], while analyses of online networks reveal Dunbar-like ceilings even in digital contexts [45,46].

These insights imply that the process of tie formation should unfold very differently when collaborative workload is a prerequisite. Collaboration demands far more time and effort than what is typically required for acquaintanceship. Academic collaboration is a clear case. Scholars must balance the work that they produce as “main contributors” with the work that they contribute as “collaborators.” Co-authoring requires sustained joint effort from all parties, spanning the entire process from project initiation to data collection, analysis, peer review, and publication. Similarly, the collaborative nature of creativity in cultural fields also necessitates considerable effort. For instance, in hip-hop music, it is a prevailing norm that rappers write their own lyrics. Consequently, even when collaborating on another rapper’s tracks, the effort invested by the featured rapper is comparable to the effort they dedicate to their own solo projects. In both contexts, actors must allocate finite time and energy across multiple projects, and the cumulative workload imposes a natural ceiling on the number of ties they can complete.

The Barabási-Albert model assumes that new actors preferentially attach to established hubs, but it does not account for the workload saturation that constrains those hubs. Social actors are therefore likely to encounter an upper limit in forming and maintaining activity-intensive ties, producing degree saturation and departures from scale-free growth.

### Preferential attachment with activity constraints: Numerical simulations

Our numerical simulations model special cases where the linear preferential attachment is contingent on the existing node’s growth constraints—particularly, an upper bound of high-fitness nodes whose capacity to accept and complete ties is finite.

Let us call the probability function in Eq 2 as $\Pi^{linear}$ because the Barabási-Albert model is known to produce α>1. We extend the linear preferential attachment model in ways that nodes’ ability to complete new ties varies when *k*(*in*) becomes large. After introducing an upper bound ϕ, Eq 2 now becomes:


$$\Pi^{threshold}(k_{i})=\frac{\min(\overset{\left(in\right)}{\underset{i}{k}},\phi )}{\sum_{j}\min(\overset{\left(in\right)}{\underset{j}{k}},\phi )}$$ (3)

Eq 3 implies that once the *k*(*in*) exceeds a certain threshold ϕ, a higher *k*(*in*) no longer enhances the likelihood of receiving a new tie. In addition to $\Pi^{threshold}(k_{i})$, we propose a “dropout” model where the degree of hubs is saturated, and they no longer accept a new tie:


$$\begin{array}{c} \Pi^{dropout}(k_{i})=\left\{ \begin{array}{cc} \frac{k_{i}^{\left(in\right)}}{\sum_{j\in L}k_{j}^{\left(in\right)}}, & k_{i}^{\left(in\right)}<\phi \\ 0, & k_{i}^{\left(in\right)}\geq \phi \end{array}\right.\; \end{array}$$ (4)

Where $L=j|j\in G,k_{j}^{\left(in\right)}<\phi$. Eq 4 is different from the threshold model in that hubs with $k^{\left(in\right)}=\phi$ is discretely unselected by a new node to connect to.

To consider noise in tie formation, we follow Refs [7,20] and introduce a tunable parameter τ that governs the degree to which the link creation is driven by preferential attachment. With a probability 1−τ, an existing node *i* is selected uniformly (randomly) with *n* nodes are present:


$$\Pi^{uniform}(k_{i})=\frac{1}{n}$$ (5)

With these three models varied by the probability functions $\Pi^{linear}$, $\Pi^{threshold}$, and $\Pi^{dropout}$, we conduct numerical simulations that, at each time step, add a new node and link it with a directed tie to an earlier added node until the graph *G* is populated with *m* nodes. Holding constant each node sending the same number of ties *d* and other initial conditions, our experimental focus is on how the distribution of the resulting in-degree sequences, $\{\overset{\left(in\right)}{\underset{i}{k}}\}=\overset{\left(in\right)}{\underset{1}{k}},\overset{\left(in\right)}{\underset{2}{k}},\cdots ,\overset{\left(in\right)}{\underset{m}{k}}$, differs by the three models. We set ϕ=100 for the threshold model and ϕ=150 for the dropout model to reflect moderate versus strict activity constraints. The value ϕ=150 corresponds to Dunbar’s number—the widely cited cognitive limit on stable social ties—while ϕ=100 represents a softer constraint in which nodes can still receive links but no longer gain additional attractiveness. Our experiment comprises 100 instances for each model. The procedures generating the synthetic network are summarized in Algorithm 1.

**Algorithm 1 Simulation algorithm to generate synthetic network.**

**Generate Synthetic Network**

**Input** τ=2/3. *d* = 3. ϕ=100 for $\Pi_{\text{threshold}}$. ϕ=150 for $\Pi_{\text{dropout}}$. *m* = 5,000.

**Output:** Directed graph *G*

1: Generate a complete directed graph *G* with 4 nodes

2: **while** |*V*(*G*)| < *m* **do**

3:  Add new node *i* to *G*

4:  **while** $\overset{\left(out\right)}{\underset{i}{k}}\leq d$ **do**

5:   **In linear model** With probability τ, select existing node *j* based on $\Pi_{\text{linear}}$; with probability 1−τ, select existing node *j* based on $\Pi_{\text{uniform}}$

6:   **In threshold model** With probability τ, select existing node *j* based on $\Pi_{\text{threshold}}$; with probability 1−τ, select existing node *j* based on $\Pi_{\text{uniform}}$

7:   **In dropout model** With probability τ, select existing node *j* based on $\Pi_{\text{dropout}}$; with probability 1−τ, select existing node *j* based on $\Pi_{\text{uniform}}$

8: **In all models:**      Add directed tie from *i* to *j*

9: **end While**

10: **end While**

11: **return** *G*

### Analysis of simulation results

Compared with the networks generated by the $\Pi^{linear}$, the networks generated by the $\Pi^{threshold}$ and $\Pi^{dropout}$ exhibit different characteristics in the tail of *k*^(*in*)^. In Table 1, we can find that the differences between these three types of synthetic networks are mainly in the nodes with the highest *k*^(*in*)^. That may make it possible for *Pr*(*k*^(*in*)^) to be more non-scale-free.

**Table 1:** Mean value of *k^(in)^* for the nodes with the most *k^(in)^* in three models with 100 instances. Standard errors in parentheses.

g	1^st^	5^th^	20^th^	50^th^	100^th^	250^th^
$\Pi^{linear}$	594.0 (8.42)	269.1 (5.65)	72.1 (0.70)	36.2 (0.26)	21.6 (0.11)	10.6 (0.05)
$\Pi^{threshold}$	293.6 (2.47)	208.7 (2.31)	78.8 (0.68)	40.3 (0.23)	24.1 (0.11)	11.4 (0.05)
$\Pi^{dropout}$	150 (0.00)	150 (0.00)	85.9 (0.34)	43.2 (0.12)	25.5 (0.05)	12.0 (0.02)

For simplicity, our analytical focus is on whether the resulting network follows a power law or lognormal distribution, since testing between the two distributions for empirical observations has been repeated across many fields over many years [47]. To examine the influence of activity constraint of nodes in the final distributions of *k*^(*in*)^ of networks, we analyze it as follows: (i) conduct a formal statistical test [2] to see whether *k*^(*in*)^ sequences of different synthetic networks fit power-law or lognormal tails or not, (ii) test if power-law can better fit to the data of the *k*^(*in*)^ sequences tails as opposed to the lognormal.

**Fig 1:**
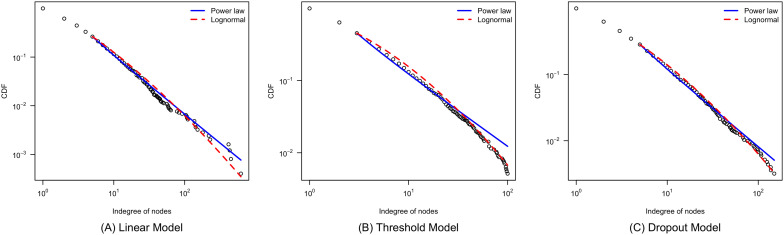
CDF of three different preferential attachment models. The cumulative distribution functions (CDFs) and their maximum likelihood power law and log normal fits on one instance of the synthetic networks generated by three different preferential attachment models.

Fig 1 presents the CDF plot of the *k*^(*in*)^ distributions of the three types of synthetic networks (we choose one instance for each type of models). We find that compared with the network generated by the linear model, the tails of the *k*_*i*_ sequences of the networks generated by the threshold model and dropout model fit the lognormal distribution better compared with the power-law distribution.

On the end *k*^(*in*)^ distribution of a G across 100 simulation instances for each preferential attachment model, we conduct a set of hypothesis tests adopted from Ref [2] (We considered the maximum entropy (ME) approach of Bee et al. [48] but opted for the Clauset-Shalizi-Newman (CSN) method because (1) the ME estimator requires solving a constrained optimizationproblem with substantial computational overload, and (2) Bee et al.’s own simulations show that “CSNperforms better than both UMPU and ME, in that it identifies (on average) a shorter power-law tailin the data, closer to the true threshold” (p.10) where UMPU refers to the uniformly most powerfulunbiased test.). Hypothesis testing is centered upon whether data is generated from which distribution: a power law, log-normal, or which one is better than the other. The results are summarized in Table 2. We find that imposing an upper bound of accepting ties, as in $\Pi^{threshold}$ and $\Pi^{dropout}$, makes the preferential attachment mechanism more likely to produce a log-normal degree distribution rather than a scale-free degree distribution. Such a difference is drastic in testing whether the log-normal distribution fits the k(in) data better than the power law (4^th^ column in Table 2). Activity constraints are effective in creating a non-scale-free distribution in 16% and 42% of the simulation instances.These findings remain stable across a wide range of threshold values: the threshold model produces similar outcomes for ϕ∈[80,150], and the dropout model for ϕ∈[140,200], confirming that our results are not sensitive to specific parameter choices (see our sensitivity check results summarized in Fig S1 Fig and S2 Fig in the Supporting Information). This result suggests that, when tie formation in a social network requires nodes’ finite capacity to accept and activate a received tie, hubs’ degree saturation may function as a mechanism to produce a non-scale-free degree distribution.

**Table 2:** Results of hypothesis testing on whether the end *k^(in)^* distribution of a synthetic network *G* is generated from a power law distribution (1^st^ column), a log normal distribution (2^nd^ column), whether the power law provides a better fit than the log normal distribution (3^rd^ column), whether the log-normal provides a better fit than the power law distribution (4^th^ column), and no statistical difference between the two (5^th^ column). Significance level at *p* < 0.05. The cell value pertains to the proportion of the 100 instances in each model. PL = Power law; LN = Log normal (We ran simulations with varying iteration counts (number of observations = 100, 500) and examined the stability of key statistics. Results show that an increase from 100 to 500 will not meaningfully change conclusions.).

	PL	LN	PL > LN	PL < LN	PL≈LN
$\Pi_{\text{linear}}$	1.0	1.0	.01	.02	.97
$\Pi_{\text{threshold}}$	.97	1.0	0	.16	.84
$\Pi_{\text{dropout}}$	.92	1.0	0	.42	.58

### Preferential attachment with activity constraints: Mathematical derivations

We now mathematically derive how the introduction of activity constraints makes certain social networks deviate from scale-free properties. Our threshold and dropout formulations can be seen as degree-indexed analogues of aging or deactivation models [33–36]. When degree increases monotonically with time, a degree-triggered cap approximates strong aging, since older nodes are also those with higher accumulated collaborations. There are similarities with aging and deactivation models, but they remain different because aging models suppress attachment with chronological age, while our model suppresses attachment with collaboration load. Similarly, dropout resembles vertex deactivation, but in our case deactivation is conditional on degree saturation rather than exogenous time or random events. This distinction matters because in empirical collaboration networks, actors of the same chronological age may differ widely in their tie load, and it is the latter that constrains further growth.

We show how activity constraints influence network formation and serve as a mechanism of non-scale-freeness by the following steps:

- Illustrate the similarities and differences between the original model, the threshold model, and the dropout model in terms of activity constraints, which helps understand the essential role of constraint mechanisms.
- Derive the degree distributions of both the threshold and dropout models, proving that the tails of these distributions do not follow the power law.
- Interrogation into the second moment of degree: We demonstrate that the networks generated by these models lack a key characteristic of scale-free networks. Specifically, for the networks generated by the threshold model or dropout model, the second moment of degree is finite when the network size approaches infinity, which is different from typical scale-free networks whose second moment of degree is infinity when the network size approaches infinity.

Here are preliminaries for some key concepts. When a network’s degree distribution follows a power law, the probability of a node having degree *k* is proportional to *k* raised to some power: $Pr_{k}\sim k^{-\gamma }$, where γ typically ranges between 2 and 3. This implies: (1) most nodes have relatively small degrees, (2) a tiny fraction of nodes may have very large degrees, and (3) in log-log coordinates, this distribution appears as a straight line.

The second moment of degree is the average of the squared degrees of all nodes in the network: $\langle k^{2}\rangle =\frac{1}{n}\sum_{1\leq i\leq n}d_{i}^{2}$. In typical scale-free networks, this value tends to infinity as the network size approaches infinity. This property relates to many important network characteristics, such as the critical threshold for spreading processes.

#### 2.5.1. Fundamental connections between the three models.

To understand the mechanism of activity constraints, we first need to recognize the fundamental connections between the origin, threshold, and dropout models. These three models essentially differ only in how activity constraints are implemented. Before activity constraints are activated, all three models follow the same network generation process: (1) new nodes join the network at a fixed rate, (2) the probability that new nodes connect to existing nodes is partly proportional to the degrees of existing nodes, and (3) each new node establishes *m* new connections.

Given that $\phi_{threshold}$ in the threshold model and $\phi_{dropout}$ in the dropout model are both much larger than the initial number of nodes *o* and the number of new connections *m*, which means the initial network and randomness cannot have much influence on the networks’ characteristics when enough nodes have been added to the networks, these models exhibit similar characteristics before constraints are triggered: $Pr(k)\sim k^{-\gamma }$.

When we let the constraint parameters approach infinity $\phi_{dropout}\to \infty$ or $\phi_{threshold}\to \infty$, both the proposed dropout model and the proposed threshold model will degenerate into the general form of preferential attachment, leading to the generated network having the same form of power law distribution with the origin model. As a result, it is precisely the introduction of activity constraints that alters the network’s fundamental properties.

#### 2.5.2. Degree distribution of threshold model.

The analysis of degree distribution in the threshold model can be divided into two phases: before and after constraint activation. Here, we focus on the behavior after constraint activation, as the degree distribution behavior before constraint activation has been discussed briefly in the above section.

Before formally deriving the model’s degree distribution, we define some notations. $P\overset{n}{\underset{v}{r}}$ means the probability density of the degree satisfying *k* = *v* in the model when the model has *n* nodes, $E(T_{v-1\to v})$ means the expected number of nodes whose degree increased from v−1 to *v* when a new node and the corresponding new links are added to the network. When the network grows sufficiently large, the degree distribution reaches a steady state: $P\overset{n}{\underset{v}{r}}=P\overset{n+1}{\underset{v}{r}}$ when n→∞.

This allows us to establish the following balance equation when n→∞ when v≠0 by considering the recursive relationship between $P\overset{n+1}{\underset{v}{r}}$ and $P\overset{n}{\underset{v}{r}}$ before and after a new node is added to networks with *n* nodes:


$$P\overset{n+1}{\underset{v}{r}}=\frac{nP\overset{n}{\underset{v}{r}}+E(T_{v-1\to v})-E(T_{v\to v+1})}{n+1}$$ (6)

Eq 6 links two different probability $P\overset{n}{\underset{v}{r}}$ and $P\overset{n+1}{\underset{v}{r}}$ together by considering the process of adding one new node to an *n*-nodes existing networks: when a new node is added to the existing networks, on average, $E(T_{v-1\to v})$ nodes will receive a new tie and then become nodes with *v* ties after the new node is added, and $E(T_{v\to v+1})$ nodes will receive a new tie and then be no more nodes with *v* ties, this means the expected number of nodes in the new networks with *v* ties can be inferred by $nP\overset{n}{\underset{v}{r}}+E(T_{v-1\to v})-E(T_{v\to v+1})$, where $nP\overset{n}{\underset{v}{r}}$ represents the expected number of nodes with *v* ties before the new node is added. As there are *n* + 1 nodes in the new network, the density of nodes with *v* ties can be calculated by the ratio between the expected number of nodes with *v* ties $nP\overset{n}{\underset{v}{r}}+E(T_{v-1\to v})-E(T_{v\to v+1})$ and the number of all nodes in the networks *n* + 1. Similarly, when *v* = 0, we have the simple variation of Eq 6, which is:


$$P\overset{n+1}{\underset{v}{r}}=\frac{nP\overset{n}{\underset{v}{r}}+1-E(T_{v\to v+1})}{n+1}$$ (7)

Here, 1 represents the exact number of new nodes with *v* = 0 ties after one node is added to the existing networks, and the remaining part in this equation is the same as Eq 6. And considering the expected number of nodes with *n* ties that will receive a new tie and have *v* + 1 nodes after one new node is added to the networks $E(T_{v\to v+1})$, we have:


$$\begin{array}{c} E(T_{v\to v+1})=\left\{ \begin{array}{cc} m(P_{1}\frac{Pr_{v}\phi }{S}+P_{2}\frac{Pr_{v}n}{n}), & \text{if }v\geq \phi \\ m(P_{1}\frac{Pr_{v}v}{S}+P_{2}\frac{Pr_{v}n}{n}), & \text{if }v<\phi \end{array}\right.\; \end{array}$$ (8)

where *m* represents the number of new links a new node can bring to the network, $S=\sum_{0\leq i\leq \phi }iPr_{i}+\sum_{\phi <i}\phi Pr_{i}$, *P*_1_ represents the probability that the new links will be added into the network following the preferential attachment mechanism, where $P_{1}+P_{2}=1$. *P*_1_ must be less than 1, otherwise no new node with *k* = 0 will receive new links. Eq 8 provides an intuitive explanation of the new degree allocation process.

Combining Eq 6 and Eq 8, we get the recursive equation between *Pr*_*k*=*v*_ and $Pr_{k=v-1}$. For simplicity, we denote *Pr*_*v*_ as *x* and $Pr_{v-1}$ as *y*, and Eq 6 can be rewritten as follows:


$$\begin{array}{c} (n+1)x=\left\{ \begin{array}{cc} nx+\frac{mP_{1}\phi y}{S}+mP_{2}y-\frac{mP_{1}\phi x}{S}-mP_{2}x, & \text{if }v>\phi \\ nx+\frac{mP_{1}(v-1)y}{S}+mP_{2}y-\frac{mP_{1}vx}{S}-mP_{2}x, & \text{if }v\leq \phi \end{array}\right.\; \end{array}$$ (9)

and this leads to the important recursive relationship between *Pr*_*k*=*v*_ and $Pr_{k=v-1}$, which is:


$$\begin{array}{c} Pr_{v}=\left\{ \begin{array}{cc} \frac{\left[\frac{mP_{1}\phi }{S}+mP_{2}\right]}{\left[1+\frac{mP_{2}\phi }{S}+mP_{2}\right]}Pr_{v-1}, & \text{if }v>\phi \\ \frac{\left[\frac{mP_{1}(v-1)}{S}+mP_{2}\right]}{\left[1+\frac{mP_{2}v}{S}+mP_{2}\right]}Pr_{v-1}, & \text{if }v\leq \phi \end{array}\right.\; \end{array}$$ (10)

As a result, we can obtain the recursive relationship between $Pr_{v-1}$ and *Pr*_*v*_, and given the value of $Pr_{v-1}$, the corresponding value of *Pr*_*v*_ can be inferred based on *m*, *P*_1_, *v* and *S*. Although it is difficult to find the analytical solution of *S* as we need to solve the high-order Eq 11, it is doable to find the numerical solution *S* of this equation, and it is known that *S* satisfies *S* < *m*, and *S* is related to m,ϕ- controlling *m*, the higher the threshold ϕ, the lower *S*.


$$\left\{ \begin{array}{c} 1=\sum_{v=0}^{\infty }Pr_{v}=Pr_{0}+t_{1}*Pr_{0}+t_{2}t_{1}Pr_{0}+\cdots +\overset{t-k}{\underset{\phi }{t}}t_{\phi -1}t_{\phi -2}\cdots t_{2}t_{1}Pr_{0},\\ S=\sum_{0\leq i\leq \phi }iPr_{i}+\sum_{\phi <i}\phi Pr_{i} \end{array}\right.$$ (11)

where $t_{\phi }=\frac{\left[\frac{mP_{1}\phi }{S}+mP_{2}\right]}{\left[1+\frac{mP_{2}\phi }{S}+mP_{2}\right]}$ and $t_{v}=\frac{\left[\frac{mP_{1}(v-1)}{S}+mP_{2}\right]}{\left[1+\frac{mP_{2}v}{S}+mP_{2}\right]}$ for *v*.

From Eq 10, as $P_{1},P_{2},S,m,\phi$ are irrelevant with *v*, we know that $\frac{Pr_{v}}{Pr_{v-1}}$ is a constant which also irrelevant with *v* when v>ϕ, suggesting that when n→∞, $Pr_{k}\sim k^{-\gamma }$ will no longer hold. Moreover, the threshold mechanism makes few nodes satisfy $k\gg \phi_{threshold}$ and more nodes satisfy $\phi_{threshold}<k$ compared to the origin model.

From Eq 10, we infer the following result that:


$$\begin{array}{c} Pr_{k}\sim \left\{ \begin{array}{cc} H^{k-\phi }, & k\geq \phi \\ k^{-\gamma_{2}}, & \phi >k \end{array}\right.\; \end{array}$$ (12)

where *H* is a constant. Eq 12 suggests that the degree distribution in the threshold model has heterogeneous characteristics above or below the threshold. Below the threshold, the network maintains power law-like behavior, while above the threshold, the distribution shows exponential decay, and this behavior fundamentally differs from typical scale-free networks.

To help illustrate the recursive relationship between $Pr_{v-1}$ and *Pr*_*v*_, we substitute $m=3,P_{1}=\frac{2}{3}$ into Eq 10, and the corresponding degree distribution follows Eq 13.


$$\begin{array}{c} Pr_{v}=\left\{ \begin{array}{cc} (1-\frac{S}{2S+2\phi })Pr_{v-1}, & \text{if }v>\phi \\ \frac{1}{2}(1+\frac{v-2}{S+v})Pr_{v-1}, & \text{if }0<v\leq \phi \\ \frac{1}{2}, & \text{if }0=v \end{array}\right.\; \end{array}$$ (13)

#### 2.5.3. Degree distribution of dropout model.

In the above section, we have proven that the degree distributions of networks following the threshold model differ from a power-law distribution. In this section, we consider the situation of degree distributions in the dropout model.

Considering the process of a new node and the corresponding ties are added to the existing network with *n* nodes, we have the same equation with Eq 6 and Eq 7 above. However, respect to the new tie allocation process $E(T_{v\to v+1})$, we have:


$$\begin{array}{c} E(T_{v\to v+1})=\left\{ \begin{array}{cc} 0, & \text{if }v\geq \phi \\ m(P_{1}\frac{Pr_{v}v}{S}+P_{2}\frac{Pr_{v}n}{n(1-Pr_{\phi })}), & \text{if }v<\phi \end{array}\right.\; \end{array}$$ (14)

Similar to the threshold model, *m* represents the number of new links a new node can bring to the network, $S=\sum_{0\leq i<\phi }iPr_{i}$ represents the “effective” sum of ties that will participate in the preferential attachment process of tie allocation, and *P*_1_ represents the probability that the new links will be added into the network following the preferential attachment mechanism. However, different from the threshold model, in the dropout model, the nodes with ϕ ties will not participate in the new ties allocation process, making Eq 14 differ from Eq 8, especially for the nodes with ϕ ties.

Combining Eq 6 and Eq 14, we get the recursive equation between *Pr*_*v*_ and $Pr_{v-1}$. For simplicity, we denote *Pr*_*v*_ as *x* and $Pr_{v-1}$ as *y*, and Eq 6 can be re-written as follows:


$$\begin{array}{c} (n+1)x=\left\{ \begin{array}{cc} 0, & \text{if }v>\phi \\ nx+\frac{mP_{1}(\phi -1)}{S}y+\frac{mP_{2}}{1-x}y, & \text{if }v=\phi \\ nx+\frac{mP_{1}(v-1)}{S}y+\frac{mP_{2}}{1-Pr_{\phi }}y-\frac{mP_{2}v}{S}x-\frac{mP_{2}}{1-Pr_{\phi }}x, & \text{if }v<\phi \end{array}\right.\; \end{array}$$ (15)

and this leads to the important recursive relationship between *Pr*_*k*=*v*_ and $Pr_{k=v-1}$, which is:


$$\begin{array}{c} Pr_{v}=\left\{ \begin{array}{cc} 0, & \text{if }v>\phi \\ \frac{1}{2}[wPr_{v-1}-\sqrt{\left(wPr_{v-1}+1{)}^{2}-4(w+z\right)}], & \text{if }v=\phi \\ \frac{w+\frac{z}{1-Pr_{\phi }}}{1+u+\frac{z}{1-Pr_{\phi }}}Pr_{v-1}, & \text{if }v<\phi \end{array}\right.\; \end{array}$$ (16)

Where $w=\frac{mP_{1}(v-1)}{S}$, $u=\frac{mP_{1}v}{S}$, and *z* = *mP*_2_. Eq 16 provides the recursive relationship between $Pr_{v-1}$ and *Pr*_*v*_, given the value of $Pr_{v-1}$, the corresponding value of *Pr*_*v*_ can be inferred.

From Eq 16, we know that the degree distributions follow Eq 17 when n→∞. There is a cut-off in the degree distribution of the dropout model when k>ϕ due to the dropout mechanism, which means that there was no “tail” in the dropout model at all when k>ϕ, and it can be found that $Pr_{k=\phi }\gg Pr_{k=\phi -1}$, which means that it is impossible for the tail of the degree distribution fits the power law distribution.


$$\begin{array}{c} Pr_{k}\sim \left\{ \begin{array}{cc} 0, & k>\phi \\ F(m,p,c), & k=\phi \\ k^{-\gamma }, & k<\phi \end{array}\right.\; \end{array}$$ (17)

Here F(m,p,ϕ) is a non-trivial function with respect to m,p,ϕ. Similarly to Eq 11, it is difficult to write the analytical solution of $Pr_{k}=\phi$ as we need to find the solution of Eq 18.


$$\left\{ \begin{array}{c} 1=\sum_{v=0}^{\phi }Pr_{v}=Pr_{0}+t_{1}*Pr_{0}+t_{2}t_{1}Pr_{0}+\cdots +Pr_{\phi },\\ S=\sum_{0\leq i\leq \phi }iPr_{i} \end{array}\right.$$ (18)

where $t_{v}=\frac{w+\frac{z}{1-Pr_{\phi }}}{1+u+\frac{z}{1-Pr_{\phi }}}$, which is consistent with Eq 16.

To help illustrate the relationship between $Pr_{v-1}$ and *Pr*_*v*_, we substitute *m* = 3, $P_{1}=\frac{2}{3}$ into Eq 16, and we have Eq 19.


$$\begin{array}{c} Pr_{v}=\left\{ \begin{array}{cc} 0, & \text{if }v>\phi \\ \frac{1}{2}[\frac{2v-2}{S}Pr_{v-1}-\sqrt{\left(\frac{2v-2}{S}Pr_{v-1}+1{)}^{2}-4(\frac{2v-2}{S}+1\right)}], & \text{if }v=\phi \\ \frac{\frac{S}{1-Pr_{\phi }}+2v-2}{(1+\frac{1}{1-Pr_{\phi }})S+2v}Pr_{v-1}, & \text{if }0<v<\phi \\ \frac{1-Pr_{\phi }}{2-Pr_{\phi }}, & \text{if }0=v \end{array}\right.\; \end{array}$$ (19)

Till now, we have provided enough evidence suggesting that both the tails of the degree distributions of the dropout model and the threshold model do not fit the power law distribution, to help illustrate the differences in the degree distribution between our threshold and dropout model and the origin model, here we provide the relationship between $Pr_{k=v-1}$ and *Pr*_*k*=*v*_ in the origin model when *m* = 3 and $P_{1}=\frac{2}{3}$, we have:


$$\begin{array}{c} Pr_{v}=\left\{ \begin{array}{cc} \frac{1}{2}(1+\frac{v-2}{3+v})Pr_{v-1}, & \text{if }0<v\\ \frac{1}{2}, & \text{if }0=v \end{array}\right.\; \end{array}$$ (20)

### Visualization of degree distributions in origin, threshold and dropout models

**Fig 2:**
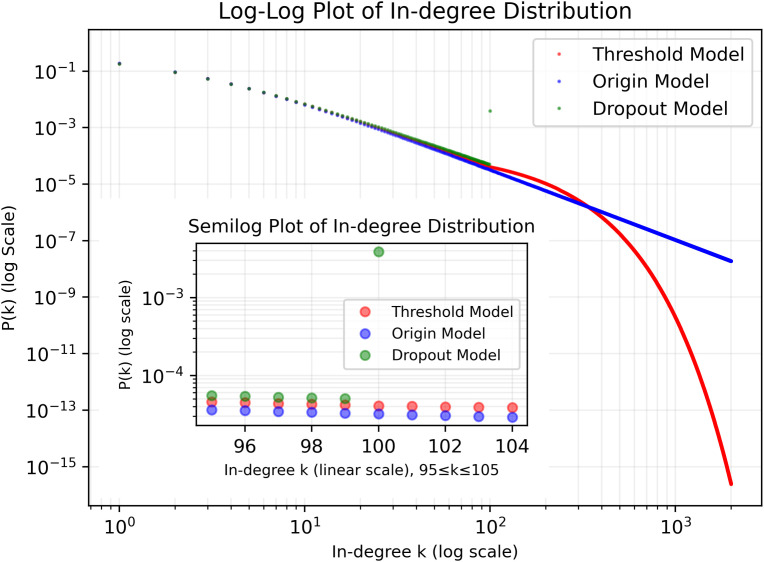
Expected Degree Distributions in the Origin, Threshold and Dropout Models (ϕ=100). The inset presents the expected degree distribution around ϕ, while the overall figure presents the expected degree distribution when k<ϕ and k>ϕ.

In Fig 2, we provide a visualization of the expected degree distributions of these three models in Eq 13, 19 and 20 when $\phi_{threshold}=\phi_{dropout}=100$.

The degree distributions of the networks generated by the threshold model and dropout model do not fit the power-law distribution: for the threshold model, its degree distribution shows exponential decay when k>ϕ, leading to the expected degree distribution not fitting a straight line in the tail when k>ϕ in log-log coordinates, which is inconsistent with the power-law distribution’s characteristics; and for the dropout model, we can notice that it does not have any nodes that have very large degrees, while the power-law distribution requires there should be a tiny fraction of nodes have very large degrees, and the degree distribution fits two different lines when k>ϕ and *k*.

In conclusion, the threshold model and dropout model are similar to the power-law distribution when *k*, which is what we expected, however, both of these models differ from the power-law distribution when k>ϕ.

#### 2.6.1. Second moment of degrees in the networks.

When a network’s degree distribution follows a power law, the probability of a node having degree k is proportional to k raised to some power: $Pr_{k}\sim k^{-\gamma }$, where γ typically ranges between 2 and 3. These networks have a finite first moment ⟨k⟩ when n→∞, however, their second and higher moments, $\langle k^{2}\rangle$, $\langle k^{3}\rangle$, go to infinity. And in our origin model, as a scale-free network, its corresponding degree exponent is 2.5, which means that $\langle k_{origin}^{2}\rangle \to \infty$ when n→∞.

However, with the degree distributions of the threshold model and dropout model provided above, it can be proven that there exist numbers $N_{1},N_{2}<\infty$ which satisfy $\langle k_{threshold}^{2}\rangle <N_{1}$ and $\langle k_{dropout}^{2}\rangle <N_{2}$, which means that the networks generated by our proposed mechanism lack the ‘scale-free’ characteristics that many scale-free networks can have- both the second moment of degrees in the dropout model and the threshold model is finite, and a number *N* that satisfy $\langle k^{2}\rangle \leq N$ when n→∞ can be found.

Before formally proving $\langle k^{2}\rangle \leq N$, we define the symbols that might be used as follows.

The network of interest has *n* nodes, and the corresponding degrees of these nodes are $d_{1},d_{2},\cdots ,d_{n}$. And we also know that according to the definition of the second moment, $\langle k^{2}\rangle =\frac{1}{n}\sum_{1\leq i\leq n}d_{i}^{2}$.

And for the dropout model, we have:


$$\begin{array}{c} \begin{array}{cc} \langle k_{dropout}^{2}\rangle & =\frac{1}{n}\sum_{1\leq i\leq n}d_{i}^{2}<\frac{1}{n}\sum_{1\leq i\leq n}\phi^{2}=\frac{1}{n}*n*\phi^{2}=\phi^{2} \end{array}\; \end{array}$$ (21)

As a result, we know that we can find a number $N_{1}=\phi^{2}$ satisfies $\langle k_{dropout}^{2}\rangle <N_{1}<\infty$, which means the second moment in the dropout model is finite.

And for the threshold model, we have:


$$\begin{array}{c} \begin{array}{cc} \langle k_{threshold}^{2}\rangle & =\frac{1}{n}\sum_{1\leq i\leq n}d_{i}^{2}=\frac{1}{n}\sum_{0\leq j\leq k_{max}}n*Pr_{j}*j^{2}\\ & =\sum_{0\leq j\leq \phi }Pr_{j}*j^{2}+\sum_{\phi <j\leq k_{max}}Pr_{j}*j^{2}\\ & <\phi^{2}+S_{k_{max}} \end{array}\; \end{array}$$ (22)

Where $S_{k_{max}}=\sum_{\phi <j\leq k_{max}}s_{j}$, and $s_{j}=Pr_{j}*j^{2}$.

To determine the finiteness of $S_{k_{max}}$, we perform the ratio test on the sequence {*s*_*j*_}, according to Eq 13, we have:


$$\begin{array}{c} \begin{array}{cc} L & =\lim_{j\to \infty }\frac{s_{j+1}}{s_{j}}=\lim_{j\to \infty }\frac{Pr_{j+1}*(j+1{)}^{2}}{Pr_{j}*j^{2}}\\ & =\lim_{j\to \infty }\frac{Pr_{j}*(1-\frac{S}{2S+2\phi })*(j+1{)}^{2}}{Pr_{j}*j^{2}}\\ & =\lim_{j\to \infty }(1-\frac{S}{2S+2\phi })[1+\frac{2}{j}+\frac{1}{j^{2}}]\\ & =\lim_{j\to \infty }(1-\frac{S}{2S+2\phi })\\ & <1 \end{array}\; \end{array}$$ (23)

Given $\lim_{j\to \infty }\frac{s_{j+1}}{s_{j}}<1$, we know that *s*_*j*_ convergences, which means $S_{k_{max}}=\sum_{\phi <j\leq k_{max}}s_{j}<\infty$. For Eq 22, we can find a number $N_{2}=\phi^{2}+S_{k_{max}}$ satisfies $\langle \overset{2}{\underset{threshold}{k}}\rangle <N_{2}<\infty$, suggesting that the second moment in the threshold model is finite, making the networks no longer scale-free.

Through these analyses provided above, we prove that activity constraints can be the mechanism making networks no longer scale-free.

## Analysis of a hip-hop collaboration network

### Fitness model

We have so far demonstrated that imposing an upper bound of accepting ties makes the preferential attachment mechanism more likely to produce a log-normal degree distribution rather than a scale-free degree distribution. This proposition, however, requires empirical evidence that actors whose *quality* is perceived to attract other actors indeed have constraints in their growth of the number of links.

The fitness model [18] incorporates the intrinsic quality of nodes (such as an individual’s social skills, contents of a web page, or scientific article) to compete for links at the expense of other nodes. In this model, the time dependence of a node’s degree depends on the fitness, $\eta_{i}$, of the node:


$$\Pi (k_{i})=\frac{\eta_{i}k_{i}}{\sum_{j}\eta_{j}k_{j}}$$ (24)

where η is chosen from the distribution ρ(η). The precise form of *Pr*(*k*) then depends on the fitness distribution ρ(η). Analytically, *Pr*(*k*) is not robust against changes in the functional form of ρ(η), such that with a choice of ρ(η) one can obtain a non-power-law distribution [18,19,49,50]. Yet $\eta_{i}k_{i}$ may be trivial, provided that $\eta_{i}$ in real-world networks very likely correlates with *k*_*i*_ (“older nodes with more ties tend to be of greater quality”). However, the precise functional form of how $\eta_{i}$ and *k*_*i*_ are correlated is nontrivial and little known in prior research. That is the empirical task undertaken in this study.

### Quality, featuring indegree, and preferential attachment in a hip-hop collaboration network

#### 3.2.1. Data.

We leverage a hip-hop collaboration network where rappers invite or are invited by one another to feature on their songs. The hip-hop collaboration network provides a useful empirical context in which (i) the directionality of a tie is clearly indexed: If there is a song “Main Artist (*i*) – SONG TITLE (feat. Invited Artist (*j*)),” then the elements *a*_*i*,*j*_ = 1 but *a*_*j*,*i*_ = 0 in an adjacency matrix *A*; (ii) artists try to engage a fellow artist whose quality, fame, or expertise may help promote their own songs; so the featuring indegree $k^{\left(in\right)}=\sum_{j=1}^{n}a_{i,j}$ signals artist’s status in the music market; and (iii) artists can face physical limits in accepting ties, unlike websites or other nodes in non-social networks because making ties means to commit to producing a song together.

We collected data from the largest music streaming platform in Korea—Melon (https://www.melon.com). To include the population of active, current hip-hop artists as completely as possible, we sampled Korean hip-hop artists who have released any song in the Rap/Hip-hop genre tab in the last two years, resulting in 3,694 focal artists (see [25] for data description). We count the artist’s featuring frequency to measure featuring in-degrees.

#### 3.2.2. Scale-freeness of a hip-hop collaboration network.

**Fig 3:**
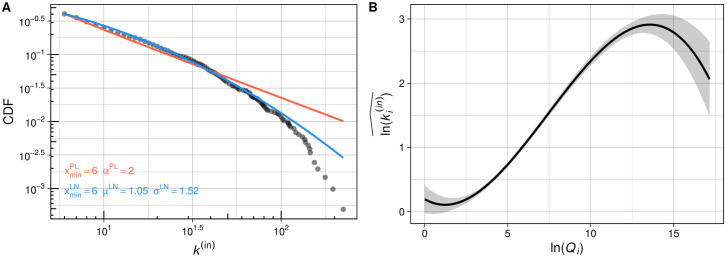
(A) The cumulative distribution functions (CDFs) and their maximum likelihood power law (red) and log normal (blue) fit for the featuring indegree of Korean hip-hop artists. (B) The estimated featuring indegree as a function of artists’ quality.

In Fig 3A, we fit power-law (red) and log-normal (blue) curves and investigated the goodness of fit, respectively. With the estimated $\hat{\alpha }=1.98$, the log-linear better fits the empirical featuring indegree data. The bootstrap hypothesis test of Ref [2] can reject the *H*_0_ for power-law (p-value:.002) but not reject the H0 for log-normal (p-value:.361). The fitted power law and log normal are compared using a likelihood ratio test. We obtain the log-likelihood ${ℒ}_{PL}$ and ${ℒ}_{LN}$ of the best fit. Their difference yields the likelihood ratio test statistic $ℛ={ℒ}_{PL}-{ℒ}_{LN}=-4.462$ (p-value<.001).

#### 3.2.3. The functional form of the quality-degree dependency.

Do high-quality artists face activity constraints in collaboration? We estimate the relationship between artists’ quality and indegree and test whether it follows a sublinear relationship: quality increases with the featuring invites but not as much to result in the appearance of super-connected nodes. We measure an artist’s quality, *Q*_*i*_, as the cumulative like counts that the artist received for all the released songs (Quality includes the count of likes for all the songs an artist has released under his or her name. This excludes the songs where he or she was featured on other artists.). Our assumption is that, in popular culture, a musician’s quality is determined by the degree to which their songs are recognized and accepted by listeners. While alternative indicators such as streaming counts or chart rankings might appear plausible, they are either unavailable or unsuitable in our context. Streaming counts are not publicly visible on Melon, and chart rankings are highly selective, with independent hip-hop artists rarely appearing in the top charts. By contrast, cumulative “likes” are publicly available, standardized across all artists, and authenticated through Melon’s identity-verification system, which allows only one like per verified account. This design makes likes a transparent, stable, and less manipulable measure of audience recognition. More broadly, in the streaming era, platform-based engagement metrics have become widely treated as shorthand for artistic quality and cultural impact—global media, for example, often highlight Spotify milestones such as Billie Eilish surpassing billions of streams as evidence of her resonance. In this sense, Melon “likes” serve as a field-appropriate quality metric in the Korean hip-hop context, capturing the same audience-driven recognition that defines cultural success in contemporary music markets.

We fit an ordinary least squares regression model that includes *Q*_*i*_ and its polynomial terms $\overset{2}{\underset{i}{Q}}$ and $\overset{3}{\underset{i}{Q}}$:


$$\overset{\left(in\right)}{\underset{i}{k}}=\gamma_{0}+\gamma_{1}Q_{i}+\gamma_{2}\overset{2}{\underset{i}{Q}}+\gamma_{3}\overset{3}{\underset{i}{Q}}+\sum W_{i}X_{i}+\epsilon_{i}$$ (25)

$\gamma_{1}$, $\gamma_{2}$, and $\gamma_{3}$ are the coefficients to capture the non-linear effects of the artist *i*’s quality on the featuring indegree $\overset{\left(in\right)}{\underset{i}{k}}$. *X*_*i*_ denotes a vector of multiple variables that might confound the relationship between *Q*_*i*_ and $\overset{\left(in\right)}{\underset{i}{k}}$, including $\overset{\left(out\right)}{\underset{i}{k}}$, active years, TV competition program participation, the total number of released songs, and prestigious crew/label membership, and *W*_*i*_ corresponds to the vector of estimated coefficients. $\gamma_{0}$ and $\epsilon_{i}$ represent the usual intercept and error terms.

We inductively determine the best functional form for the quality variable predicting $\overset{\left(in\right)}{\underset{i}{k}}$ by comparing models (Table 3). The model-free result (Models 1–3) indicates that the cubic term increases the overall goodness-of-fit (adjusted R^2^), with all their coefficients being significantly different from 0. This polynomial effect remains robust against the inclusion of other control variables (Model 5). In Fig 3B, We plotted the $\hat{\overset{\left(in\right)}{\underset{i}{k}}}$ a function of *Q*_*i*_, which indicates that the estimated line departs from linear in a concave downward manner for high-end hip-hop artists. This result suggests that high-quality artists do receive more ties by starring at their fellow rappers’ songs but not at the level expected by their quality—especially top rappers whose degree could be a determining factor on whether the whole distribution approximates a power law.

**Table 3:** Ordinary least squares regression models predicting artist’s featuring indegree.

	(1)	(2)	(3)	(4)	(5)
Total likes	0.269	0.256***	−0.148***	0.261***	−0.035
	(0.006)	(0.022)	(0.054)	(0.021)	(0.051)
Total likes (Quad)		0.001	0.066***	−0.007***	0.041***
		(0.001)	(0.008)	(0.001)	(0.008)
Total likes (Cubic)			−0.003***		−0.002***
			(0.0004)		(0.0003)
Active years				0.015***	0.012**
				(0.005)	(0.005)
Number of songs				−0.001***	−0.001***
				(0.0002)	(0.0002)
Number of collaboration songs				0.008***	0.009***
				(0.001)	(0.001)
Prestigious label membership				0.337***	0.331***
				(0.037)	(0.036)
Crew membership				0.799***	0.767***
				(0.071)	(0.071)
TV program appearances				0.317***	0.292***
				(0.047)	(0.047)
Intercept	−0.543***	−0.503***	0.196*	−0.488***	0.022
	(0.036)	(0.072)	(0.111)	(0.068)	(0.105)
Observations	3,694				
R^2^	0.377	0.378	0.389	0.453	0.459
Adjusted R^2^	0.377	0.377	0.388	0.452	0.458

*Note:* * *p* < 0.1; ** *p* < 0.05; *** *p* < 0.001.

## Analysis of an academic collaboration network

Here, we examine the quality-degree dependency within a different empirical context: academic collaboration. The scaling of scale-freeness in academic networks is highly plausible. Networks formed by citation patterns exhibit an infinite degree bound [51–53] whereas networks formed through collaboration patterns are constrained by authors’ commitments. Academic publication data makes it possible to investigate how different tie formation processes that are differed by the commitment levels (co-authoring vs. citation) unfold within the same domain.

### SciSciNet data

SciSciNet is a large-scale open data lake specifically designed for research in the science of science [26]. This lake was constructed based on the dataset of the latest and final edition of the Microsoft Academic Graph (MAG), released on December 6, 2021. It encompasses over 134 million scientific publications authored by 134 million researchers across 19 different fields, up to the year 2021. SciSciNet is specifically tailored for the science of science research, therefore providing good accuracy for our interested quantities (co-author degree and citation counts), while other repositories like CiteSeerX, AMiner, Crossref, and OpenAlex have broader focuses on various scientific disciplines and academic social networks.

### Analytical strategy

By definition, preferential attachment processes assume variations in nodal growth within a specific timeframe for a defined population. Researchers enter and exit the field at different times and durations, making it challenging to observe all these dynamics using unstructured publication data. To address this, we need boundary conditions to define a population and analyze degree distributions within it.

Our strategy involves defining a subset of authors as a cohort, based on their shared “debut” timing in a specific field, and evaluating the growth of coauthor degree inequality years later within that cohort. For example, we might examine psychologists who first published in the early 1990s and analyze how their coauthor degree distributions evolved over the next 30 years. This approach aligns with the scope of our dataset, as we observe (only) current article citation counts and author quality metrics (e.g., h-index) and can track down (unique) author degree at a specific time point using the recorded year of publication. So, we fix the current time as a fixed time point while varying the first timing of entry into the field.

Without this cohort-based approach, it becomes difficult to account for the time-related factors that influence coauthor behavior and citation dynamics. These factors include: (i) The increasing number of journal outlets and publications in recent years; (ii) The broader prevalence of academic collaboration in contemporary times; and (iii) The natural decay of an author’s active publishing period and a paper’s academic impact over time. Given these complexities, aggregating authors with differing active periods without temporal boundaries would lead to inaccurate analyses.

It is important to highlight the field information used in this study. SciSciNet relies on the fields of study records from the Microsoft Academic Graph (MAG). MAG employs a hierarchical taxonomy to classify fields of study, capturing the relationships between research areas and their subfields. This taxonomy spans a total of 19 fields. To construct a cohort in a field, we follow these steps:

- Identify authors who have ever published in a journal within a defined field.
- Subset the cohort to include authors whose first publication year falls within a specific period, such as a 5-year span from 1990 to 1994.
- Calculate each author’s total unique degree (number of distinct collaborators) and the cumulative count of their coauthor connections across all of their papers.

Notably, this approach does not rely on within-field author degrees or restrict papers strictly to within-field classifications, as publication and collaboration often cross field boundaries. Instead, we adopt a broad definition of author-field association (“ever published in a field”), consistent with the dataset settings employed by Broido and Clauset [7].

We use the h-index as a proxy for the quality of an author. The h-index is widely recognized for its ability to combine both the quantity and impact of a researcher’s work into a single metric. Unlike other bibliometric indicators, such as the total citation count, which can be disproportionately influenced by a single highly-cited paper, or the publication count, which does not account for the impact of the work, the h-index provides a balanced view by considering both aspects simultaneously. Additionally, it addresses some limitations of alternative metrics like the g-index, which gives excessive weight to highly-cited papers, and the i10-index, which only counts papers with at least ten citations, potentially overlooking the broader citation distribution. We acknowledge that the H-index has limitations, such as sensitivity to career length and field-specific citation practices, and it does not fully capture the impact of a small number of breakthrough works. However, it remains the most widely adopted and consistently available bibliometric indicator at scale, making it especially suitable for large-N comparative analyses like ours. Alternative field-normalized or composite indices are not consistently available for our dataset, whereas the H-index provides a pragmatic, continuous, and widely interpretable proxy for scholarly quality.

### Results

For illustrative purposes, we selected a cohort that debuted in the early 1990s (1990–1994) in the field of psychology (We begin with the 1990-1994 cohort because earlier cohorts suffer from incomplete bibliometric coverage and higher rates of retirement, making them less comparable. A 30-year observation window is sufficient to capture the full arc of academic careers, from early growth to saturation.). Psychology is a social science discipline that is large enough to be representative and exhibits active co-authoring patterns (in contrast to disciplines like philosophy or history) while being less prone to hyper-authorship (as seen in fields like physics or medicine). The cohort that first published during 1990–1994 provides approximately 30 years of observations in our data. In the United States, on average, it takes around 18–24 years to achieve the rank of full professor from the start of Ph.D. studies. This includes an estimated 6 years for completing a Ph.D., followed by 12–18 years in academic ranks (typically 6 years as an assistant professor and an additional 6–8 years as an associate professor). Therefore, selecting the 1990–1994 cohort allows for a sufficiently long period to observe variations in scholarly careers while also focusing on individuals who remain actively engaged in academia.

We take a random sample of 2,500 authors in the cohort as a focal analysis subset. The challenge of extremely large datasets is that even very small effects can become statistically significant due to the sheer sample size. This can lead to results that are technically significant but not necessarily meaningful in a practical sense. Taking a random sample is a straightforward way to reduce the dataset to a manageable size while preserving representativeness and generalizability. This approach also helps visualization and eases computational burdens.

**Fig 4:**
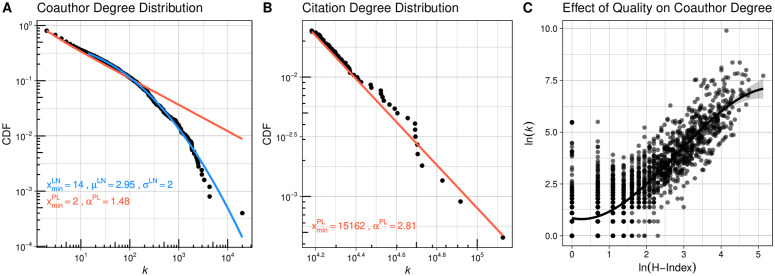
An illustrative case for psychology authors. Coauthor degree **(A)**, citation degree distribution **(B)**, and the relationship between H-index and coauthor degree **(C)** for a random sample (n = 2,500) of the cohort whose first year of publication in psychology journals fell in the period of 1990-1994, with their publication records tracked until 2023 by SciSciNet data.

Overall, we find that coauthor degree follows a non-scale free distribution while citation degree approximates a scale-free distribution (Fig 4A and 4B). For Fig 4A, similar to the test applied for the aforementioned analysis of the hip-hop featuring network, we fit both a lognormal curve (blue) and power law curve (red) and conducted a log-likelihood test after fixing the *X*_*min*_. We find the log-likelihood ${ℒ}^{PL-LN}=-5.824(p\text{-value}<\mathrm{.001})$, which suggests that log-normal better represents the observed co-author degree distribution. But the citation degree distribution appears to be a power law distribution, with its α is greater than 2 (Fig 4B).

How does coauthor degree vary by the quality of authors? To determine the functional form, we fit a linear, quadratic, and cubic form of regression that regress coauthor degree as a function of author’s H-index. Then, we compared their R^2^ to determine one that best explains the variation of the coauthor degree. The cubic model had the highest R^2^ (Table 4), and we visualized its effect plot in Figure 4C. The negative coefficient (−0.381) for the cubic term suggests the presence of an S-shaped curve. Its implication for the behavior of the high-quality authors in the tail is that, as h-index increases, the coauthor degree does not grow linearly and infinitely; rather, it departs from linear in a concave downward manner.

**Table 4:** OLS regression models predicting log coauthor degree for psychology authors first published in 1990–1994, with functional form comparisons (Models 1–3), calendar year and career age fixed effects (Model 4), and all-field specification (Model 5).

Dependent Variable:	Log Coauthor Degree (Psychology)				Log Coauthor Degree
	Linear	Quadratic	Cubic	Year FE	Field FE
	(1)	(2)	(3)	(4)	(5)
Constant	$0.564^{***}$	$1.109^{***}$	$1.315^{***}$	$2.364^{***}$	$1.480^{***}$
	(0.027)	(0.035)	(0.042)	(0.194)	(0.047)
H-Index	1.215***	0.242***	-0.442***	-0.339***	-0.166***
	(0.015)	(0.047)	(0.089)	(0.053)	(0.011)
H-Index (Quad)		0.253***	0.702***	0.562***	0.527***
		(0.012)	(0.051)	(0.031)	(0.007)
H-Index (Cubic)			-0.075***	-0.057***	-0.057***
			(0.008)	(0.005)	(0.001)
Career Age				-0.030***	0.008***
				(0.006)	(0.002)
Yr 2000 FE				-1.010***	-0.148***
				(0.142)	(0.034)
Yr 2010 FE				-0.500***	-0.031·
				(0.080)	(0.019)
Field FE	No	No	No	No	Yes
R^2^	0.735	0.776	0.784	0.708	0.672
Adj. R^2^	0.734	0.776	0.783	0.707	0.672
Num. obs.	2,500	2,500	2,500	7,500	142,500

^***^p < 0.001; ^**^p < 0.01; ^*^p < 0.05; ·*p < 0.1*

In Model 4, we extend the analysis by calculating each author’s cumulative coauthor degree at multiple interim calendar year snapshots (2000, 2010), in addition to the most recent year (2022). This design allows us to follow the same cohort of authors over time and to estimate pooled regressions with both career age and calendar year fixed effects. By incorporating interim snapshots, we explicitly account for temporal dynamics of network evolution and discipline-wide shocks that could otherwise confound the observed relationship. We set Year 2022 as the reference category, so the coefficients for 2000 and 2010 are negative, indicating that collaboration levels in earlier years were significantly lower than in 2022. This specification absorbs discipline-wide shocks such as the rise of multi-authorship in psychology during the 2000s and 2010s. Crucially, the cubic terms remain highly significant, showing that the S-curve persists net of these period effects. Career age enters with only a modest negative coefficient, suggesting that the nonlinearity is not simply driven by longer exposure time.

Finally, Model 5 extends the specification to all 19 fields (field fixed-effects terms included but not shown). The quadratic and cubic terms remain highly significant, and the S-curve is evident across disciplines. This broader specification demonstrates that the nonlinear quality–collaboration relationship is structural rather than field-specific, suggesting the generality of the pattern beyond psychology.

**Fig 5:**
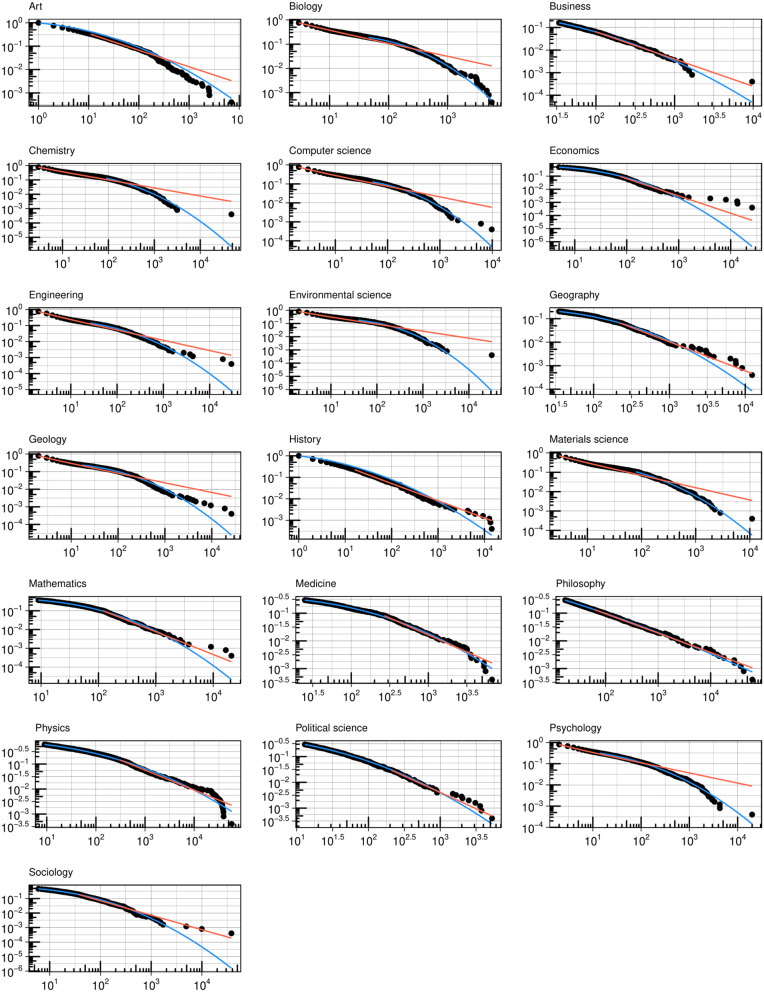
Coauthor degree distribution for a random sample (n = 2,500) of the cohort debuted in 1990-1994 by fields.

**Fig 6:**
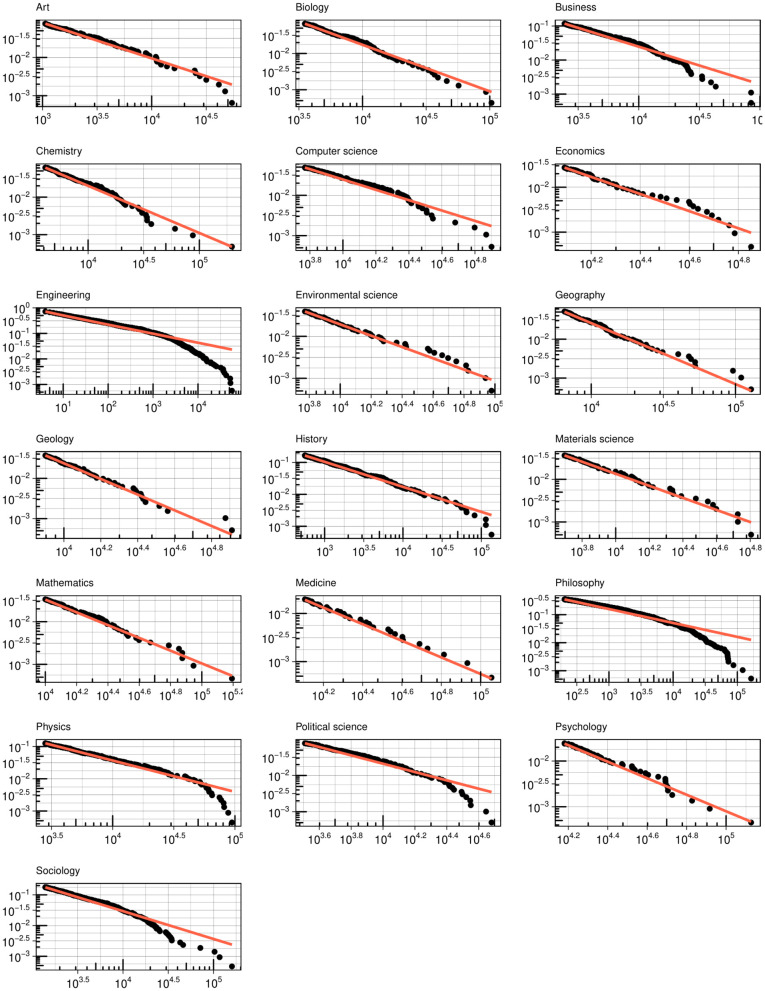
Citation degree distribution for a random sample (n = 2,500) the cohort debuted in 1990-1994 by fields.

**Fig 7:**
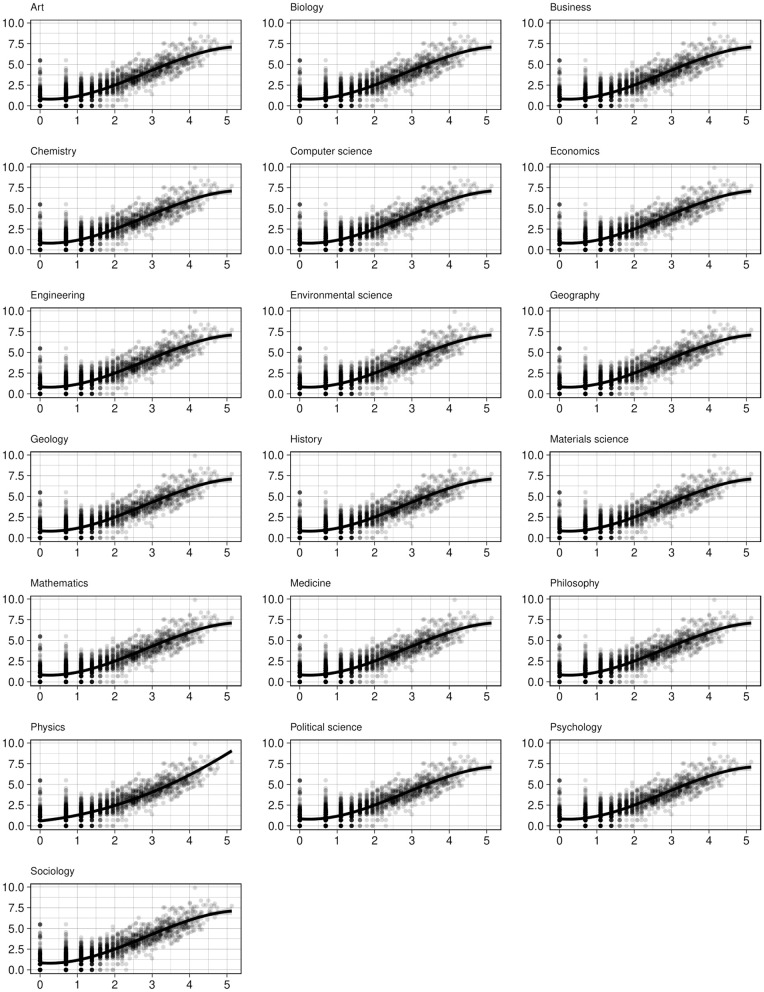
Coauthor degree as a function of H-index for a random sample (n = 2,500) of the cohort debuted in 1990-1994 by fields.

How general are our findings beyond psychology? We conducted the same analysis for other fields and plotted the coauthor and citation degree distributions (see Fig 5 and Fig 6, respectively). For coauthor degrees, a log-normal curve provides a better fit for most fields, with a few exceptions. In fields where the number of authors per paper is relatively low (e.g., sociology, philosophy), rare hubs occupy a larger proportion of degrees. For citation degrees, as expected, a power-law curve effectively represents the distribution for most fields. In Fig 7, we present effect plots for the Ordinary Least Squares (OLS) model that best explains the variation in author degree, using the H-index as the predictor. With the exception of physics—where the quadratic model yields the highest R^2^—we find that the S-shaped curve produced by the cubic model is the most plausible functional form for the quality-degree dependency. This suggests the finite growth of coauthor degrees for authors at the tail of the distribution.

How do activity constraints stabilize over time? Activity constraints are inherently time-dependent: when researchers first enter a field, they have greater capacity to establish collaboration ties. However, as time progresses, their ability to form new ties tends to diminish. This insight suggests that activity constraints are likely less prominent in the early generation of researchers compared to later generations who have had sufficient time of exposure in the field.

**Fig 8:**
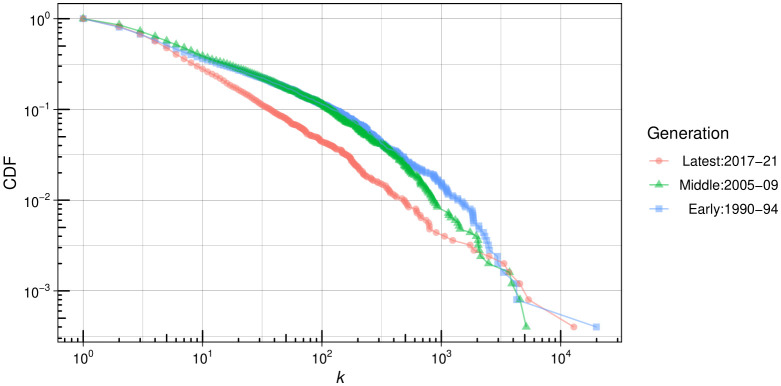
Coauthor degree distribution by author cohorts in psychology.

The wealth of SciSciNet dataset enables us to investigate how coauthor degree distributions stabilize over time. We created three cohorts based on the timing of their first publication: 1990–1994, 2005–2009, and 2017–2021. Fig 8 presents the coauthor degree distributions for early (average 28.5 years of exposure), middle (average 13.5 years), and latest generations of psychologists (average 2.5 years). Interestingly, the degree distribution for the latest generation appears relatively closer to a scale-free pattern. As time progresses (from middle to early generations), the density in the intermediate degree range becomes increasingly pronounced; however, this trend is less evident in the tail range. While researchers’ coauthor ties expand, this growth is uneven. This illustrates how coauthor degree distributions stabilize over time due to activity constraints that suppress the tail, gradually approaching a log-normal distribution. In the Supporting Information, we include plots for the middle (Fig S3 Fig, S4 Fig, S5 Fig) and latest (Fig S6 Fig, S7 Fig, S8 Fig) generations across all fields, which largely exhibit similar stabilization patterns.

## Discussion

This study has proposed an activity-constraint approach where the finite tie-completing capacity of hubs or high-degree actors conditions scale-freeness in social networks. Our numerical simulations illustrated that imposing an upper bound of accepting ties makes the preferential attachment mechanism more likely to produce a log-normal degree distribution rather than a scale-free degree distribution. Then, we mathematically derived that the two activity-constrained hub behaviors (threshold and dropout) necessarily make the degree distribution deviate from scale-free. Our empirical analysis demonstrates the possibility that such an upper bound as activity constraints can indeed exist in the domains of hip-hop music collaboration and academic publishing practices. In both hip-hop featuring and academic coauthor networks that require investment of social actors to complete ties, we found that the degree distributions are heavy-tailed but non-scale-free—unlike the constraint-free citation indegree distribution that follows a power law. Further, we revealed a non-linear, S-shaped relationship between actors’ quality (total like counts for hip-hop artists and H-index for authors) and degree, which substantiated the quality-degree dependency: the degree saturation levels exist for hubs.

These findings carry a direct implication for the body of studies adjudicating power-law degree distribution in empirical networks. Scale-freeness in social networks can crucially hinge upon whether tie formation among social actors necessitates nodes’ activity or commitments. Back to the influential paper of Broido and Clauset [7] who found that scale-free is often not a better model for most social networks, their datasets are all subject to activity constraints similar to our hip-hop collaboration network: movie actors, academic collaboration, and terrorists. Not all social networks require nodes’ commitments to complete a tie. For instance, receiving a follow on a social media platform does not consume time and energy and thus produces a scale-free distribution [54]. In a similar vein, scaling of scale-freeness in academic networks is very likely: A network created by citation patterns has an infinite degree bound [51–53] whereas a network created by collaboration patterns is bound by authors’ commitments. We claim that work searching for empirical scale-free networks should differentiate tie-formation types since activity constraints as a mechanism, in part, determine finite or infinite growth [8,9], which then conditions how its degree distribution approaches a power law or other alternatives.

This paper stands as the first empirical investigation of the functional form of the fitness correction [18] of the Barabási-Albert Model [1]. Despite its long-standing insight that the shape of degree distribution can depend on the distribution of nodal attributes (fitness or quality), work has remained only theoretical perhaps due to the lack of empirical settings where fitness is clearly measured and preferential attachment likely underlies the logic of making connections between nodes. Our empirical context of hip-hop and academic collaboration not only offers empirical measurement of fitness (listener-evaluated quality and H-index) and degrees (featuring directed ties and coauthor ties) but also reveals their non-linear relationship. That is, the quality-degree dependency is indeed influenced by hubs being saturated with excessive connections. This finding is counter-intuitive against a common linear prediction that older nodes with more ties tend to be of greater quality. Thus, this study implies that ρ(η) can indeed have a non-trivial consequence on *Pr*(*k*).

Our results also speak to prior models that suppress hub growth, such as aging, memory, and deactivation frameworks [33–36]. These approaches similarly predict deviations from pure scale-free structure, but they do so by assuming that nodes lose attractiveness with time, retain only a finite memory of past nodes, or eventually become inactive. Our activity-constraint model is still different: suppression arises not from chronological age or stochastic state changes, but from collaboration load. This distinction is empirically meaningful. In our data, actors of the same age or tenure can differ widely in their degree saturation, and it is this workload-linked ceiling that explains why collaboration networks deviate from scale-free growth. Hence, our findings complement existing hub-suppression models while highlighting a mechanism that is especially salient for activity-intensive ties.

Sociological research has long recognized that humans face cognitive and temporal limits in sustaining relationships. Dunbar’s number highlights a ceiling on stable social ties [15], while ego-network surveys consistently find only a handful of close confidants [37–39]. Classic theories of tie strength [13], social foci [40], and cumulative advantage [41] implicitly acknowledge that effortful ties are costly, and Simmel’s early account of urban life already noted the burden of excessive contacts [42]. Subsequent empirical work has illuminated these insights. For example, studies of email and mobile phone communication show that individuals have a finite “social capacity” and reallocate attention when new ties are added [43,44], while analyses of online social networks reveal Dunbar-like ceilings even in digital contexts [45,46]. Research on brokerage and tie decay likewise demonstrates that maintaining many connections is fragile and resource-intensive [55,56].

Yet these findings have largely remained at the ego-network level. Our study extends this line of work by formalizing finite tie-completion capacity as a generative mechanism and demonstrating how the micro-level burden of maintaining relationships aggregates into macro-level degree distributions that stabilize into saturated inequality patterns rather than unbounded scale-free growth. In doing so, we also refine sociological accounts of cumulative advantage: while high-status actors do attract more ties, their growth is bounded by activity constraints, producing inequality that is substantial but not unbounded. More broadly, our framework draws on core ideas from transaction cost theory [19,20], which emphasizes the coordination and effort costs inherent in repeated exchanges; we show that analogous “social transaction costs” operate in collaboration-intensive networks, limiting hub expansion and shaping the emergent degree distribution.

We wish to sound a note of caution as far as generalizing our findings is concerned. The empirical scope of hip-hop collaboration is confined to a Korean context. The unique features of our data (quality and degree measurements) contributed to a test of the functional form of the quality-degree dependency, but at a cost; we are left with the question of whether the results derived from the unique context can generalize to other networks that require nodes’ commitments to complete a tie. Hence, future studies should address whether and how activity constraints come into play in different contexts.

In conclusion, this study demonstrates that activity constraints—conceptualized as collaborative workload—constitute a generative mechanism that limits hub growth and produces non-scale-free degree distributions in social networks. By combining simulations, mathematical derivations, and empirical analyses of hip-hop and academic collaboration, we show that high-quality actors encounter saturation points that temper preferential attachment. Recognizing the role of collaborative workload highlights why some social networks deviate from scale-free patterns and points to the importance of differentiating tie-formation types when adjudicating degree distributions.

## Supporting information

S1 FigPercentage of synthetic network *G* which fitting the log normal distribution better than the power law distribution (100 threshold model instances for each ϕ value).(TIFF)

S2 FigErcentage of synthetic network *G* which fitting the log normal distribution better than the power law distribution (100 dropout model instances for each ϕ value).(TIFF)

S3 Fig Coauthor degree distribution for a random sample (n = 2,500) of the cohort debuted in 2005–2009 by fields. (TIFF)

S4 Fig Citation degree distribution for a random sample (n = 2,500) the cohort debuted in 2005–2009 by fields. (TIFF)

S5 Fig Coauthor degree as a function of H-index for a random sample (n = 2,500) of the cohort debuted in 2005–2009 by fields. (TIFF)

S6 Fig Coauthor degree distribution for a random sample (n = 2,500) of the cohort debuted in 2017–2021 by fields. (TIFF)

S7 Fig Citation degree distribution for a random sample (n = 2,500) the cohort debuted in 2017–2021 by fields. (TIFF)

S8 Fig Coauthor degree as a function of H-index for a random sample (n = 2,500) of the cohort debuted in 2017–2021 by fields. (TIFF)
