# Supplementary figures and images for: Activity constraints and the emergence of non-scale-free networks: Evidence from hip-hop and academia
Source: PLoS One. 2026 Mar 30;21(3):e0345862. doi: 10.1371/journal.pone.0345862 (PMC13035169; doi:10.1371/journal.pone.0345862)

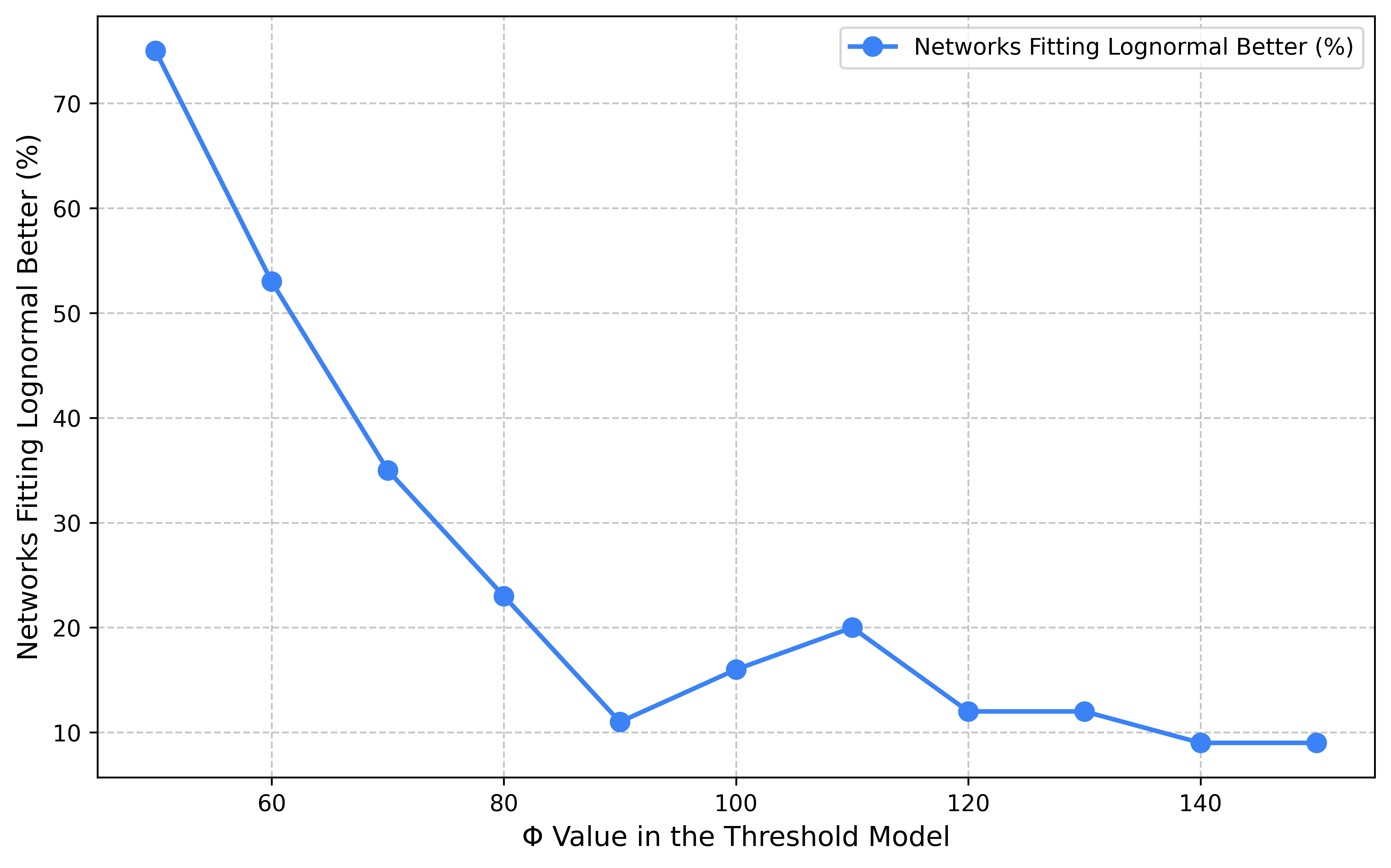

Supplement: S1 Fig — (TIFF) [file pone.0345862.s001.tiff]

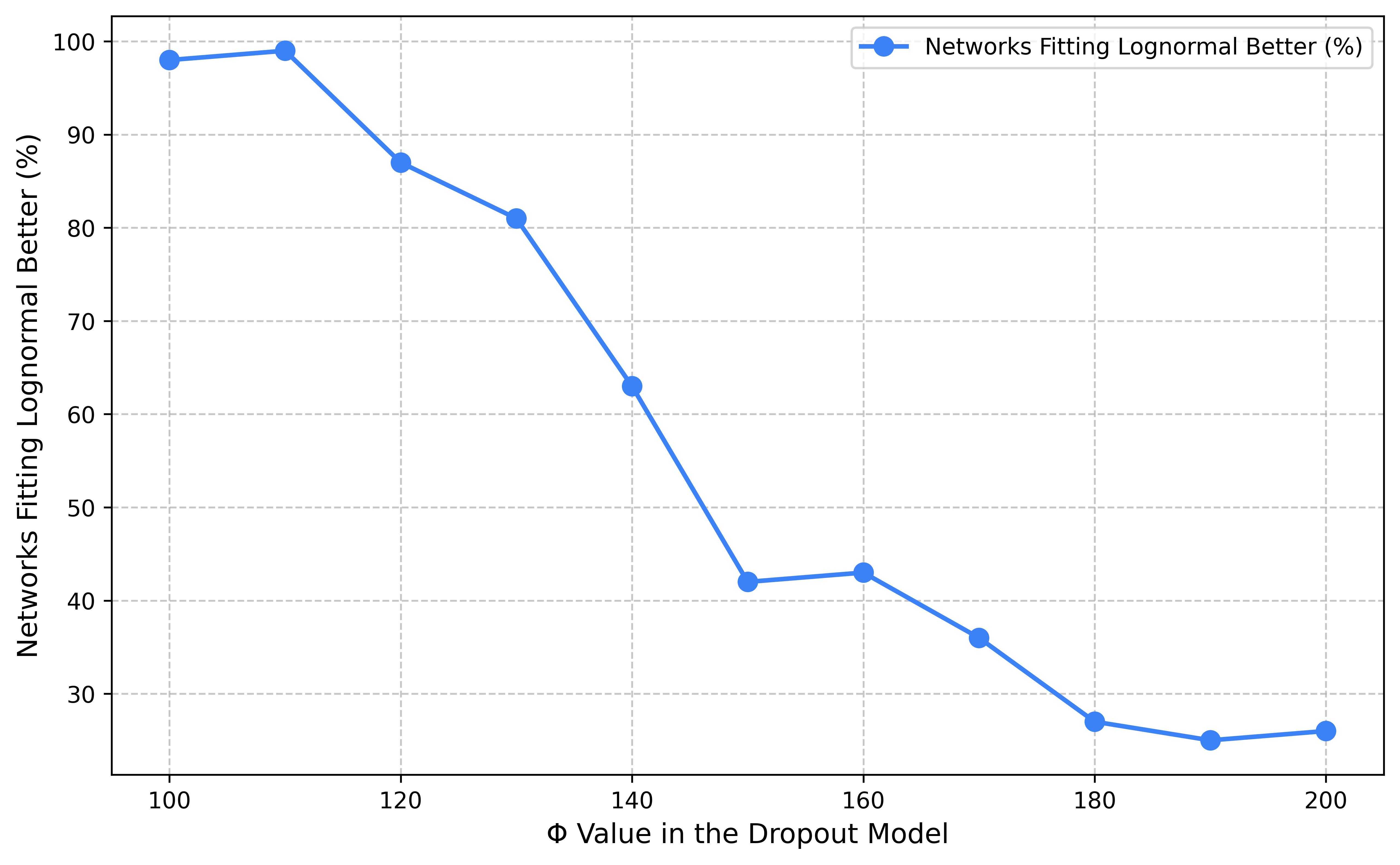

Supplement: S2 Fig — (TIFF) [file pone.0345862.s002.tiff]

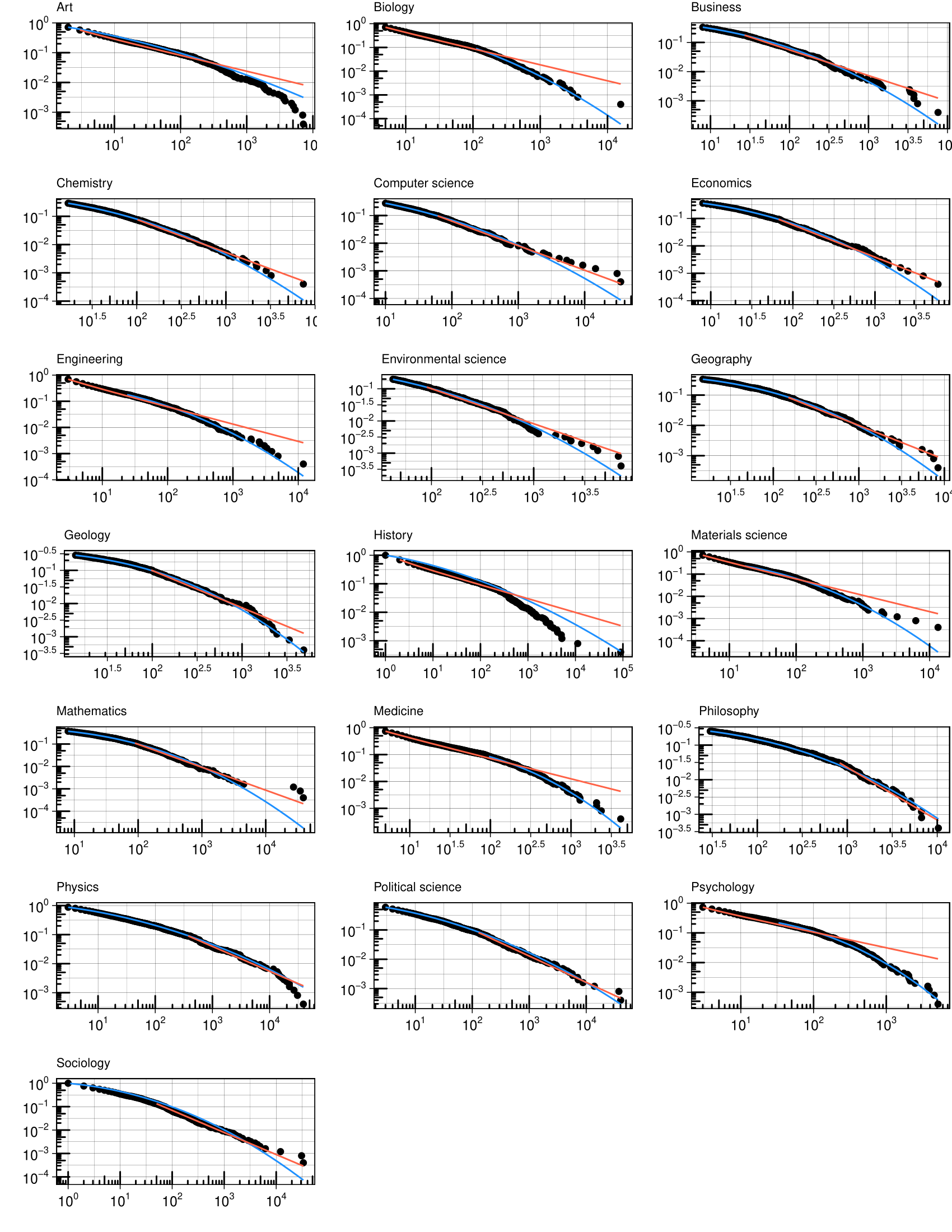

Supplement: S3 Fig — (TIFF) [file pone.0345862.s003.tiff]

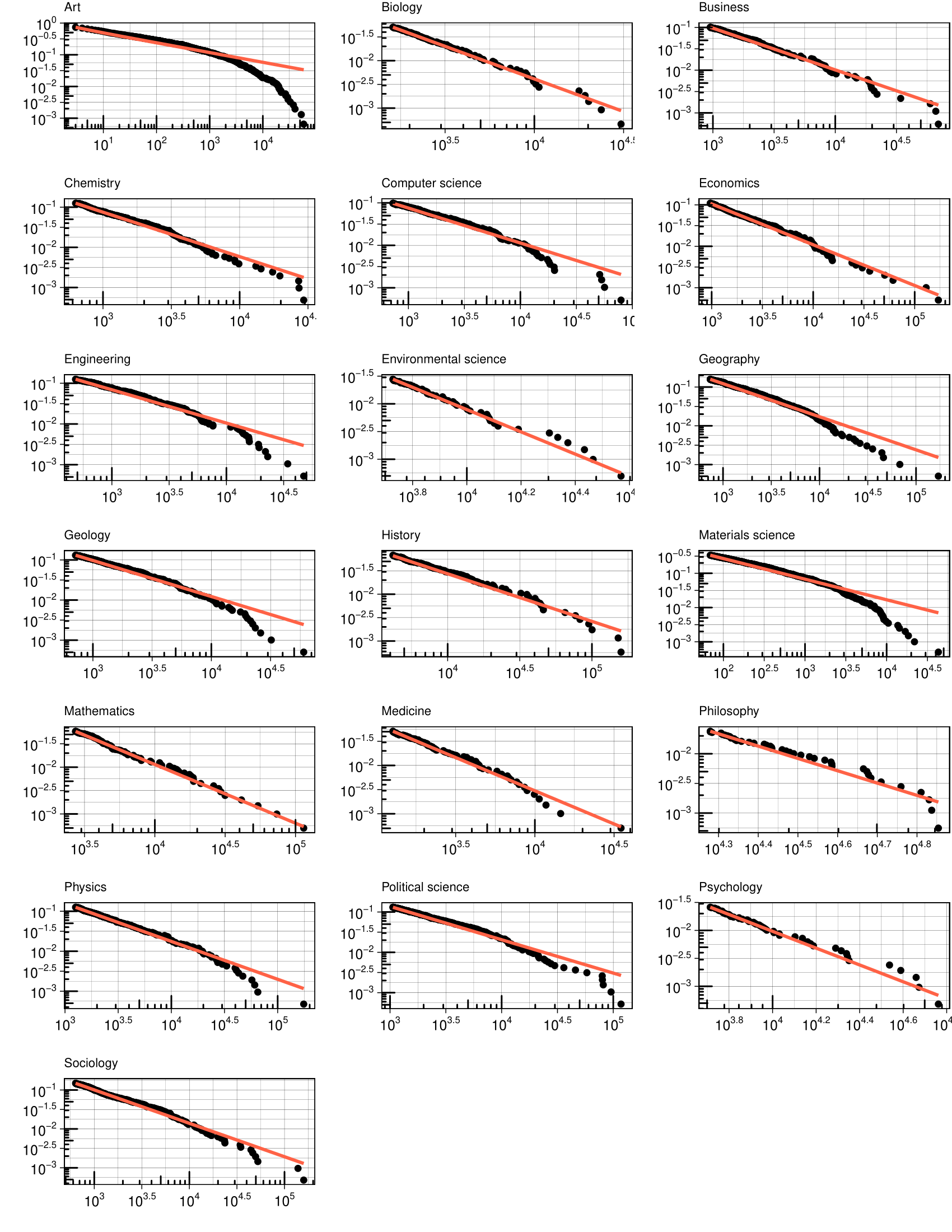

Supplement: S4 Fig — (TIFF) [file pone.0345862.s004.tiff]

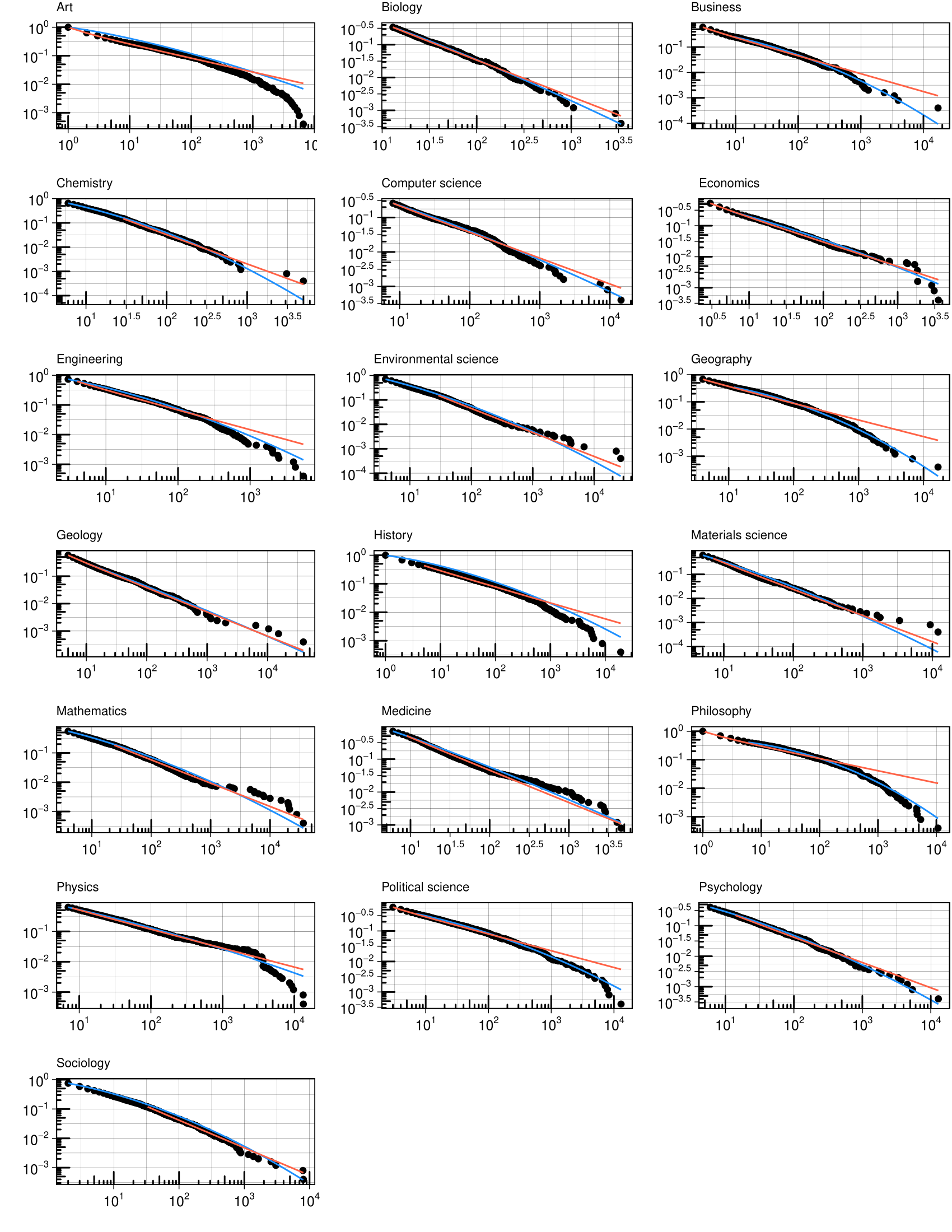

Supplement: S6 Fig — (TIFF) [file pone.0345862.s006.tiff]

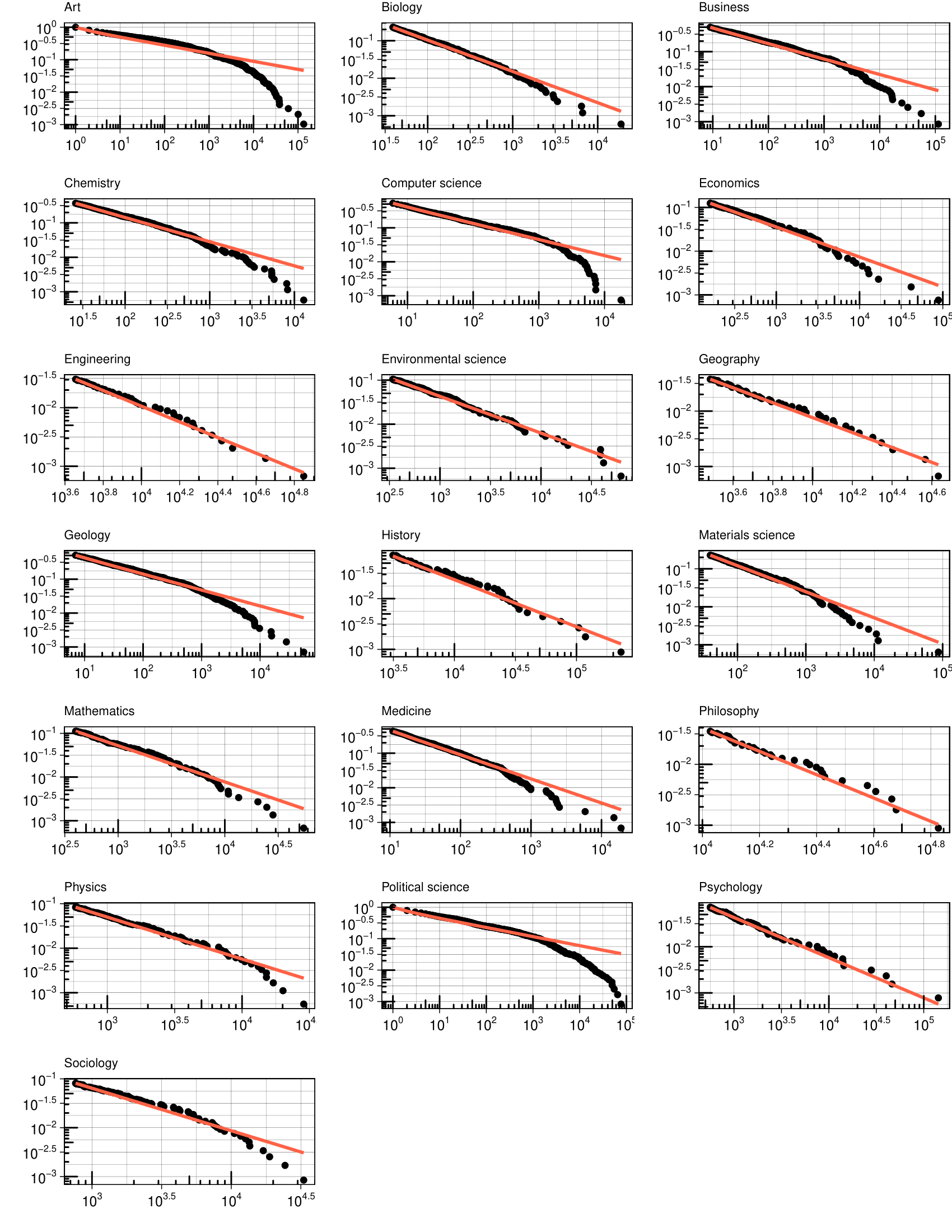

Supplement: S7 Fig — (TIFF) [file pone.0345862.s007.tiff]
